# Organization and replicon interactions within the highly segmented genome of *Borrelia burgdorferi*

**DOI:** 10.1371/journal.pgen.1010857

**Published:** 2023-07-26

**Authors:** Zhongqing Ren, Constantin N. Takacs, Hugo B. Brandão, Christine Jacobs-Wagner, Xindan Wang

**Affiliations:** 1 Department of Biology, Indiana University, Bloomington, Indiana, United States of America; 2 Department of Biology, Stanford University, Stanford, California, United States of America; 3 Sarafan ChEM-H Institute, Stanford University, Stanford, California, United States of America; 4 Howard Hughes Medical Institute, Stanford, California, United States of America; 5 Illumina Inc., 5200 Illumina Way, San Diego, California, United States of America; Centre National de la Recherche Scientifique, FRANCE

## Abstract

*Borrelia burgdorferi*, a causative agent of Lyme disease, contains the most segmented bacterial genome known to date, with one linear chromosome and over twenty plasmids. How this unusually complex genome is organized, and whether and how the different replicons interact are unclear. We recently demonstrated that *B*. *burgdorferi* is polyploid and that the copies of the chromosome and plasmids are regularly spaced in each cell, which is critical for faithful segregation of the genome to daughter cells. Regular spacing of the chromosome is controlled by two separate partitioning systems that involve the protein pairs ParA/ParZ and ParB/Smc. Here, using chromosome conformation capture (Hi-C), we characterized the organization of the *B*. *burgdorferi* genome and the interactions between the replicons. We uncovered that although the linear chromosome lacks contacts between the two replication arms, the two telomeres are in frequent contact. Moreover, several plasmids specifically interact with the chromosome *oriC* region, and a subset of plasmids interact with each other more than with others. We found that Smc and the Smc-like MksB protein mediate long-range interactions on the chromosome, but they minimally affect plasmid-chromosome or plasmid-plasmid interactions. Finally, we found that disruption of the two partition systems leads to chromosome restructuring, correlating with the mis-positioning of chromosome *oriC*. Altogether, this study revealed the conformation of a complex genome and analyzed the contribution of the partition systems and SMC family proteins to this organization. This work expands the understanding of the organization and maintenance of multipartite bacterial genomes.

## Author summary

Genomes are highly organized in cells to facilitate biological processes. *Borrelia burgdorferi*, an agent of Lyme disease, carries one linear chromosome and more than twenty plasmids in what is known as one of the most segmented bacterial genomes. How the different replicons interact with each other is unclear. Here we investigate the organization of this highly segmented genome and the protein factors that contribute to this organization. Using chromosome conformation capture assays, we determined the interactions within the chromosome, between the chromosome and plasmids, and between the plasmids. We found that the two telomeres of the linear chromosome interact with each other; a subset of plasmids interact with the chromosomal replication origin region; and a subset of plasmids preferentially interact with one another. Finally, we revealed that two structural maintenance of chromosomes (SMC) family proteins, Smc and MksB, promote long-range DNA interactions on the chromosome, and the two partition systems, ParA/ParZ and ParB/Smc, contribute to chromosome structure. Altogether, we characterized the conformation of a complex genome and investigated the functions of different genome organizers. Our study advances the understanding of the organization of highly segmented bacterial genomes.

## Introduction

*Borrelia burgdorferi* causes Lyme disease, the most prevalent vector-borne infectious disease in Europe and North America [1,2]. Although the *B*. *burgdorferi* genome is only ~1.5 Megabase pairs in size, it includes one linear chromosome and more than 20 plasmids (circular and linear) and is, to our knowledge, the most segmented bacterial genome [3–6]. Recently, using fluorescence microscopy to visualize loci on the chromosome and 16 plasmids, we found that *B*. *burgdorferi* contains multiple copies of its genome segments per cell, with each copy regularly spaced along the cell length [7].

In bacteria, the broadly conserved *parABS* partitioning system plays an important role in the segregation of chromosome and plasmids [8–15]. ParA dimerizes upon ATP binding and non-specifically binds to the DNA [16–19]. Centromeric ParB proteins bind to the *parS* sequences scattered around the origin of replication and spread several kilobases to nearby regions, forming a nucleoprotein complex [20–25]. The ParB-DNA nucleoprotein complex interacts with DNA-bound ParA-ATP dimers and stimulates the ATPase activity of ParA, leading to the release of ParA from the DNA and the formation of a ParA concentration gradient along the nucleoid [12, 15, 17, 26]. It is thought that repeated cycles of ParA and ParB interaction and release, together with the translocating forces from elastic chromosome dynamics [27–30] or the chemical ParA gradient [31, 32], promote the segregation of the two newly replicated ParB-origin complexes from one another [27, 29]. In addition, ParB plays a separate role in recruiting the broadly conserved SMC complex onto the chromosomal origin region [13, 14]. Once loaded, SMC complexes move away from the loading sites and typically tether the two replication arms together, facilitating the resolution and segregation of the two sister chromosomes [33–35].

We discovered that in *B*. *burgdorferi*, the segregation and positioning of the replication origin (*oriC*) of the multicopy chromosome require the concerted actions of the ParB/Smc system and a newly discovered ParA/ParZ system [7]. ParZ, a centromere-binding protein, substitutes ParB to work with ParA and plays a major role in chromosome segregation [7]. Although *B*. *burgdorferi* ParB does not appear to partner with ParA, it is still required to recruit Smc to *oriC*. Smc in turn contributes to *oriC* positioning [7]. Overall, these previous findings advanced our understanding of *oriC* segregation in *B*. *burgdorferi*. However, the information on the organization of the bulk of the chromosome and the interactions among the various genome segments in this bacterium is still lacking.

Chromosome conformation capture assays (Hi-C) have significantly advanced our understanding of bacterial genome folding and interactions [34, 36–41]. Along bacterial genomes, short-range self-interacting domains called chromosome interaction domains (CIDs) have been observed and are shown to be dictated mostly by transcription, with domain boundaries correlating with highly transcribed genes. In bacteria that contain the canonical SMC complex, the two replication arms of the chromosome are juxtaposed together, whereas bacteria that only encode SMC-like MukBEF and MksBEF proteins do not show inter-arm interactions [37, 39].

More recent efforts have begun to reveal the genome conformation of bacteria containing multiple replicons. In *Agrobacterium tumefaciens*, the origins of the four replicons are clustered together, which regulates DNA replication and drives the maintenance of this multipartite genome [41, 42]. Similarly, the two origins of *Brucella melitensis* chromosomes also showed frequent interactions [43]. In *Vibrio cholerae*, the origin of Chromosome 2 (Ch2) interacts with the *crtS* region on Chromosome 1 (Ch1) for replication control, and the terminus regions of Ch1 and Ch2 interact for coordinated replication termination and terminus segregation [40, 44]. These findings suggest that multipartite genomes harness inter-replicon interactions as a mechanism for replication regulation and genome maintenance. In this study, we aimed at understanding how *B*. *burgdorferi* organizes its ~20 replicons and how the partitioning proteins and Smc homologues contribute to genome organization.

## Results

### The organization of the linear *B*. *burgdorferi* chromosome

**Fig 1:**
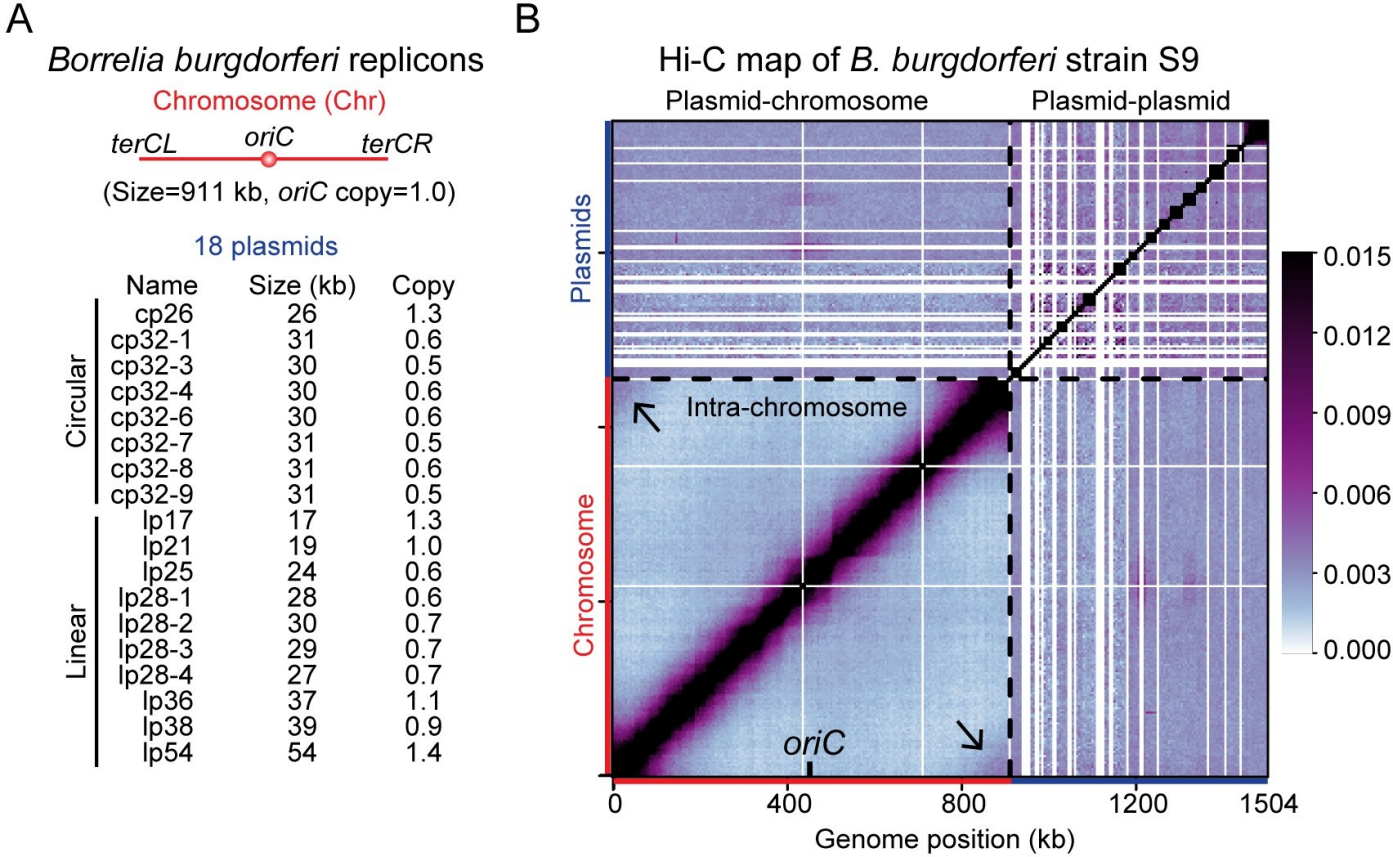
Genome-wide organization of *B*. *burgdorferi* replicons. **(A)** The *B*. *burgdorferi* S9 wild-type strain has one linear chromosome (Chr), eight circular plasmids, and ten linear plasmids. The replication origin of the chromosome is labeled as *oriC*. The sizes (in kb) and relative copy numbers of the plasmids are listed. The copy numbers of each plasmid were previously measured using whole genome sequencing analysis [7], and were shown relative to the copy number of *oriC*. **(B)** Normalized Hi-C interaction map showing interaction frequencies for pairs of 5-kb bins across the genome of *B*. *burgdorferi* strain S9. The x- and y-axes show genome positions. The chromosome and the plasmids are indicated by red and blue bars, respectively. *oriC* is labeled on the x-axis. The boundaries between the chromosome and the plasmids are indicated by black dotted lines. The white lines indicate the presence of repetitive sequences omitted during sequence mapping. The black arrows point to the interactions between the telomere regions. The plasmids are ordered alphabetically from cp26 to lp54, from left to right on the x-axis and bottom to top on the y-axis. The whole map was divided into four regions: the lower left region shows intra-chromosomal interactions, the upper left and lower right regions show plasmid-chromosome interactions, and the upper right region represents plasmid-plasmid interactions. We used the same convention for all whole-genome Hi-C and downstream analyses in this study. The color scale depicting Hi-C interaction scores in arbitrary units is shown at the right. The same Hi-C map with a different color scale is shown in **S1 Fig**.

To determine the organization of the highly segmented genome of *B*. *burgdorferi*, we performed chromosome conformation capture (Hi-C) on exponentially growing cultures of the infectious, transformable strain S9, hereafter used as our wild-type (WT) strain (**S1 Table** and **Figs 1A, 1B, and S1**). Hi-C experiments measure the frequency of DNA contacts captured by formaldehyde, which is a one-carbon crosslinker that covalently links protein-protein, protein-DNA, and DNA-DNA when these molecules are in spatial proximity [45]. A high frequency of contact in a Hi-C map indicates that the DNA pieces are either in physical contact or in spatial proximity, which may happen on their own or be mediated by protein factors. In this study, we refer to “high frequency of contact between the DNA pieces in the Hi-C maps” as “interactions” for simplicity.

After mapping the reads and plotting the data, we observed many white lines on the Hi-C map, especially in regions corresponding to the plasmids (**Fig 1B**). These white lines indicated the presence of repetitive sequences on the affected replicons, which were omitted during sequence mapping. The genome-wide Hi-C interaction map (**Fig 1B**) has four distinct regions: an intra-chromosomal interaction map in the lower left quadrant, a plasmid-chromosome interaction map with identical, mirrored copies in the upper left and lower right quadrants, and a plasmid-plasmid interaction map in the upper right quadrant. The chromosome displayed strong short-range interactions as shown on the primary diagonal (**Fig 1B**, lower left quadrant). To better present the short-range interactions on the chromosome, we plotted the Hi-C data in a different color scale (**S1 Fig**). Similar to what has been reported in other bacteria [34, 36–38], chromosome interaction domains (CIDs) were present along the chromosome (**S1A Fig**), with the strongest CIDs boundaries largely correlated with highly transcribed genes revealed by RNA-seq performed in a different study [46] (**S1B Fig**). Interestingly, a secondary diagonal representing inter-arm interactions was absent from the Hi-C map (**Figs 1B and S1**, lower left quadrant). This was unexpected as *B*. *burgdorferi* encodes an Smc protein homolog and all Smc-carrying bacteria tested so far display inter-arm interactions on the chromosome [34, 36, 38, 39, 41, 47, 48]. Notably, although *B*. *burgdorferi* contains a homolog of the ScpA subunit of the SMC complex, it does not encode the other subunit, ScpB [3]. Thus, the absence of the Smc-ScpAB holo-complex might explain the absence of chromosome arm alignment in *B*. *burgdorferi* (see Discussion). Additionally, the two ends of the linear chromosome, the left and right telomeres (*terCL* and *terCR*), displayed a high frequency of contact (**Fig 1B**, black arrows in lower left quadrant). It is unclear whether *terCL* and *terCR* regions were physically interacting through specific factors, or some unknown properties of these chromosome ends increased the probability of contact between these two DNA regions. In addition, since *B*. *burgdorferi* is polyploid [7], we do not know whether the interacting *terCL* and *terCR* were located on the same chromosome or on adjacent chromosome copies.

### Interactions between the chromosome and 18 plasmids

**Fig 2:**
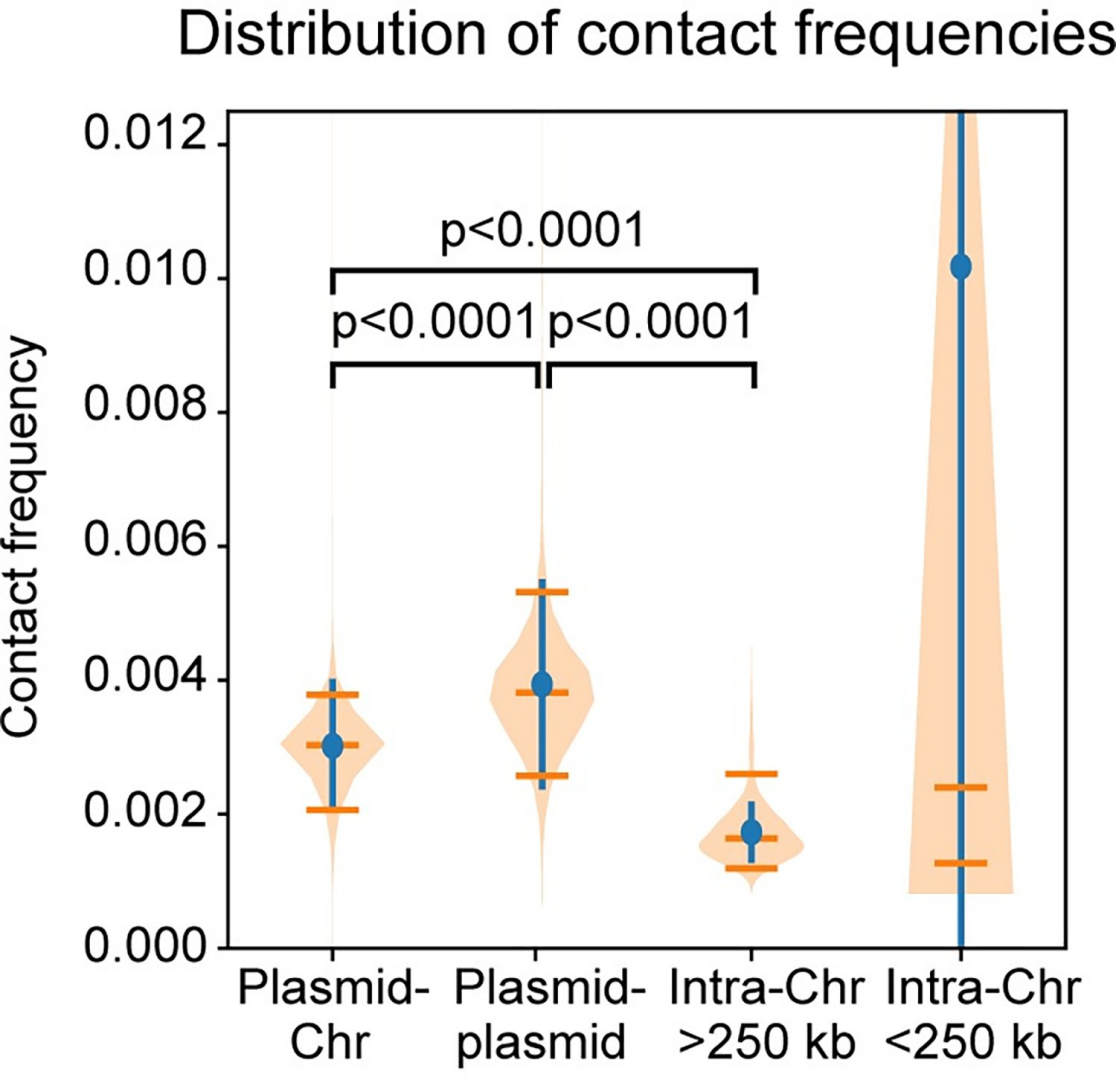
Hi-C contact frequencies for different types of interactions. Distributions of Hi-C contact frequencies measured for different types of interactions are shown as violin plots. Blue lines indicate standard deviations of the values. Orange lines indicate the median, 5^th^ and 95^th^ percentile of the data. The *p-*values were computed using a Mann-Whitney U test. All comparisons were done for data binned at 5-kb resolution.

**Fig 3:**
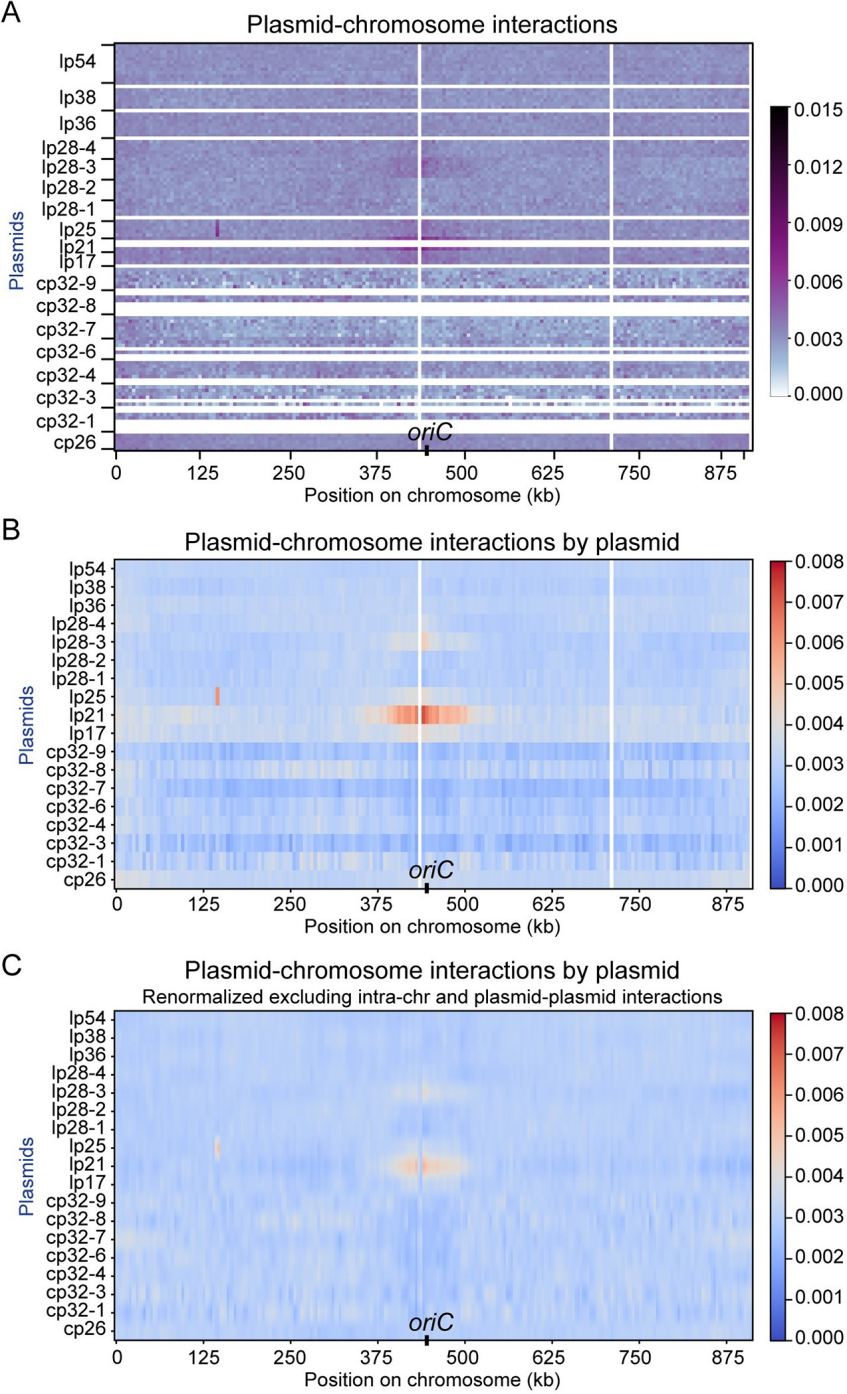
Plasmid-chromosome interactions. **(A)** Enlarged Hi-C map of plasmid-chromosome interactions in WT *B*. *burgdorferi* strain S9 from **Fig 1B**. The x-axis shows positions on the chromosome, and the y-axis shows the plasmids with their relative lengths. The white lines indicate repetitive sequences omitted during sequence mapping. *oriC* is labeled on the x-axis. The color scale depicting Hi-C interaction scores in arbitrary units is shown at the right. We note that on plasmid lp25 of WT *B*. *burgdorferi* strain S9, the *bbe02* gene was disrupted by a P_*flaB*_*-aadA* streptomycin resistance cassette. Therefore, there were two copies of P_*flaB*_, one on lp25 and one at the endogenous chromosomal locus at ~150 kb. The B31 genome sequence used for Hi-C mapping contained only the endogenous copy of P_*flaB*_. Thus, short-range interactions on lp25 involving the ectopic copy of P_*flaB*_ artifactually appeared as interactions between lp25 and the chromosome at ~150 kb. **(B)** The calculated interaction scores between each plasmid and chromosome locus. The Hi-C interaction scores in consecutive bins were summed according to each plasmid before plotting. The plot shows averaged data of two replicates. The x-axis indicates the genome position on the chromosome. The y-axis specifies the different plasmids. The color scale depicting interaction scores in arbitrary units is shown at the right. We note that these values were calculated from (**A**), which was part of **Fig 1B**. The data were normalized including all the interactions in the genome (i.e. intra-chromosomal, plasmid-chromosome and plasmid-plasmid interactions). **(C)** Renormalized plasmid-chromosome interactions following iterative correction to remove the contributions of intra-chromosomal and plasmid-plasmid interactions (see Materials and methods). The data were normalized such that each row had the same total score, and each column had the same total score.

Qualitatively, plasmid-chromosome interactions (**Figs 1B and S1**, upper left and lower right quadrants) were weaker than short-range interactions within the chromosome (**Figs 1B and S1**, the primary diagonal of the lower left quadrant), but were stronger than long-range interactions within the chromosome (**Figs 1B and S1,** outside of the primary diagonal on the lower left quadrant). We plotted the distribution of these types of interaction frequencies and found that the differences were statistically significant (**Fig 2**). To better show the plasmid-chromosome interactions (**Fig 3A**), we analyzed the interaction of each plasmid with each 5-kb bin on the chromosome by adding up the interaction scores that belonged to the same plasmid (**Fig 3B**). Interestingly, a subset of the linear plasmids, namely lp17, lp21, lp25, and lp28-3, showed higher contact frequency with the chromosome, especially in the *oriC* region compared with the rest of the chromosome (**Fig 3B**). We also observed that cp32-3, cp32-7, cp32-9 had overall lower interactions with the chromosome seen as “blue stripes” in **Fig 3B**, which was correlated with their higher plasmid-plasmid interactions (see below). To examine the plasmid-chromosome interactions without the influence of intra-chromosomal and plasmid-plasmid interactions, we renormalized the data by iterative correction (see Materials and methods) on **Fig 3B** and generated **Fig 3C**. While this renormalization removed the blue stripes seen in **Fig 3B**, the positive interactions between the four plasmids (lp17, lp21, lp25 and lp28-3) and *oriC* were still evident albeit less intense (**Fig 3C**). The plasmid-*oriC* interactions observed by Hi-C are reminiscent of the origin clustering interactions mediated by centromeric proteins in *A*. *tumefaciens*, which are critical for the replication and maintenance of the secondary replicons in that bacterium [41, 42]. Notably, the plasmid-chromosome interactions observed here are weaker than those observed in *A*. *tumefaciens*, and only four out of 18 plasmids showed these specific interactions with the chromosome, thus the biological function of these interactions is unclear (see Discussion).

### Plasmid-plasmid interactions

**Fig 4:**
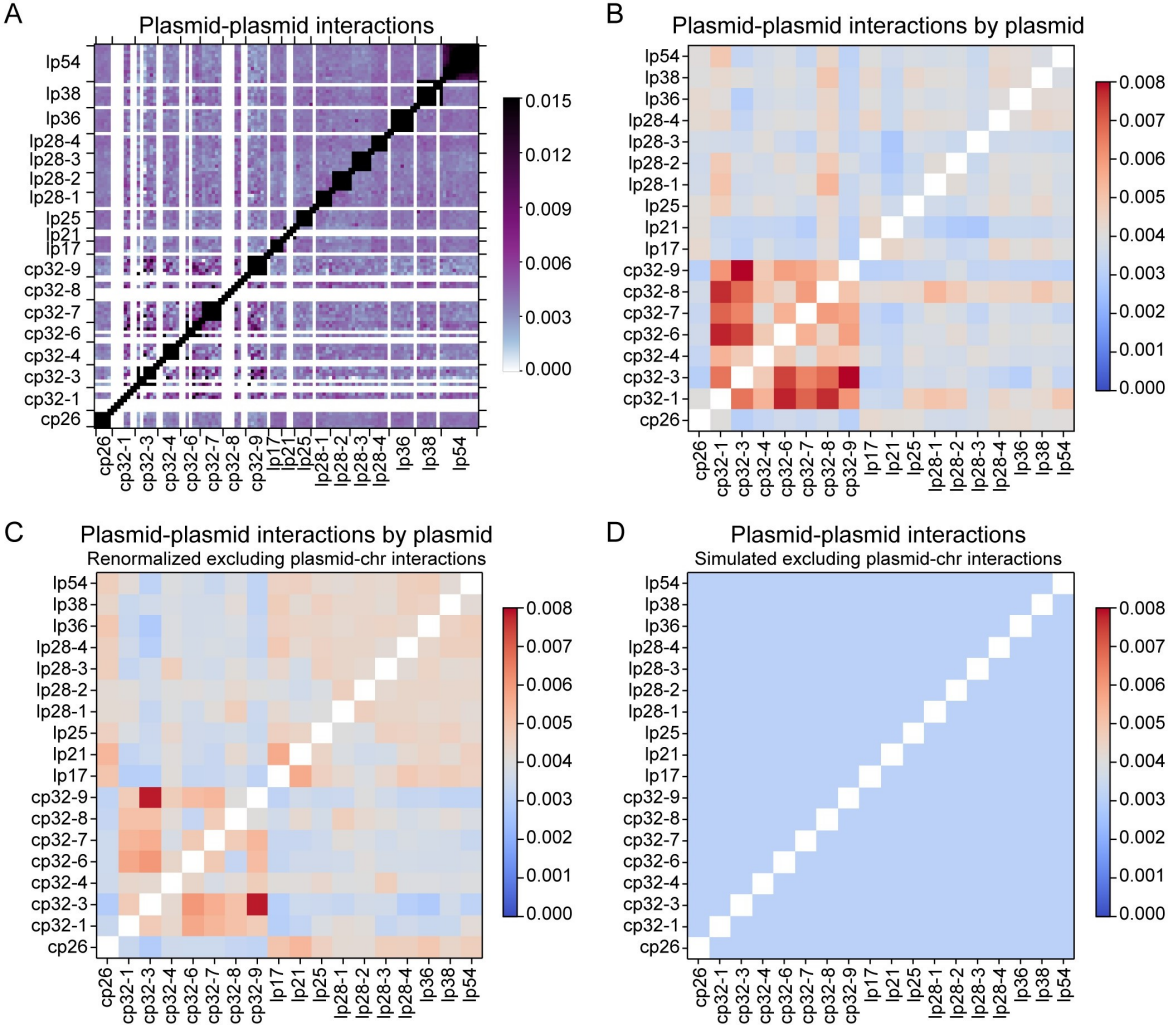
Plasmid-plasmid interactions. **(A)** Enlarged Hi-C map of plasmid-plasmid interactions in WT *B*. *burgdorferi* strain S9 from **Fig 1B**. The x- and y-axes show the plasmids with their relative lengths. The white lines indicate repetitive sequences omitted during sequence mapping. The color scale depicting Hi-C interaction scores in arbitrary units is shown at the right. **(B)** The calculated interaction scores between each pair of plasmids. The Hi-C interaction scores in consecutive bins were summed according to each plasmid prior to plotting. The plot shows averaged data of two replicates. The color scale depicting interaction scores in arbitrary units is shown at the right. We note that these values were calculated from (**A**), which was part of **Fig 1B**. The data were normalized including all the interactions in the genome (i.e. intra-chromosomal, plasmid-chromosome and plasmid-plasmid interactions). **(C)** Renormalized plasmid-plasmid interactions following iterative correction to remove the contributions of plasmid-chromosome interactions (see Materials and methods). The data were normalized such that each row had the same total score, and each column had the same total score. **(D)** The simulated interaction frequencies between plasmids based on random collisions accounting for plasmid copy numbers and plasmid sizes (see Materials and methods). The data went through iterative correction in the same way as the experimental data shown in (**C**). The simulated maps before iterative correction or after iterative correction but in a finer color scale can be found in **S2A Fig**.

Plasmid-plasmid interactions are depicted in the upper right quadrant of the Hi-C map (**Figs 1B and S1**) and appeared stronger than plasmid-chromosome interactions (**Fig 1B**, upper left quadrant, and **Fig 2**) and long-range interactions within the chromosome (**Fig 1B**, outside of the primary diagonal on the lower left quadrant, and **Fig 2**). The primary diagonal of the plasmid-plasmid interaction quadrant showed that each plasmid formed an interaction domain on its own (**Fig 4A**). We note that the sizes of the 18 plasmids range from 17 kb to 54 kb [3, 4] (**Fig 1A**) and that many plasmids have repetitive sequences omitted during Hi-C mapping (**Fig 4A**). Therefore, our Hi-C map with a bin size of 5 kb does not have high enough resolution to describe detailed intra-plasmid interactions.

To better examine the interactions between every two plasmids, we recalculated the interaction frequencies by adding up interaction scores that belonged to the same plasmid (**Fig 4B**). To remove the influence of plasmid-chromosome interactions, we renormalized the data by iterative correction (see Materials and methods) on **Fig 4B** to obtain **Fig 4C**. These analyses revealed higher interactions among the seven cp32 plasmids (cp32-1, cp32-3, cp32-4, cp32-6, cp32-7, cp32-8, cp32-9) (**Fig 4B and 4C**). To a lesser degree, the circular cp26 plasmid and the ten linear plasmids interacted more among themselves than with the cp32 plasmids (**Fig 4C**). The sizes of the plasmids range from 17 to 54 kb (**Fig 1A**). Their copy number had been previously determined by microscopy and whole genome sequencing, which ranged from 0.5 to 1.4 relative to the copy number of the *oriC* locus [7] (**Fig 1A**). To test whether the sizes and copy numbers of the plasmids might contribute to plasmid-plasmid interactions, we used these numbers to simulate the plasmid-plasmid interaction frequencies, assuming that all the plasmids were randomly interacting with each other and were freely diffusing in the cytoplasm (see Materials and methods for simulation details). Before any corrections, our simulations showed that plasmids that have a bigger size or a higher copy number interacted more with other plasmids (**S2A and S2B Fig**, top panels). However, these preferential interactions did not show up after our standard procedure of iterative corrections which were also applied to the experimental Hi-C maps [49] (**S2A and S2B Fig**, middle panels, **Fig 4D**), unless we used a very fine color scale (**S2A and S2B Fig** bottom panels). Thus, the preferential interactions between plasmids we observed in our experiment (**Fig 4B and 4C**) could not be explained solely by random plasmid-plasmid interactions after plasmid size and copy number differences were accounted for. Since repetitive sequences within the plasmids were removed during mapping, we believe that these higher-than-expected interactions observed in our experiment are genuine and not due to erroneous mapping or normalization. The molecular mechanism for plasmid-plasmid interactions remains to be determined.

### Clustering analysis of *smc* and *par* mutants

The highly conserved SMC family proteins and the DNA partitioning proteins are central players in bacterial chromosome organization and segregation [50–53]. *B*. *burgdorferi* has a canonical Smc protein, encoded by gene *bb0045*, as well as an MksB protein, encoded by gene *bb0830*, but lacks the genes encoding the accessory proteins ScpB, MksE, and MksF [3]. Additionally, *B*. *burgdorferi* employs two partition systems for the positioning of its multicopy *oriC* loci: ParB/Smc and ParA/ParZ [7]. In our previous study, we built a collection of mutants carrying the following gene deletions: *ΔparB*, *ΔparS*, *ΔparBS*, *ΔparA*, *ΔparZ*, *ΔparAZ*, *ΔparAZBS*, or *Δsmc* [7]. In these strains, the genes of interest were disrupted and replaced with a gentamycin or kanamycin resistance gene. A control strain CJW_Bb284 was also built, which had the gentamycin marker inserted in a non-coding region located between the convergently-oriented *parZ* and *parB* genes, in an otherwise WT *parAZBS* locus. We have previously shown that the mutant strains have similar growth rates compared with the S9 WT and control strains, except for the *ΔparAZBS* mutant, which grows slower [7]. Quantitative imaging has also indicated that all of these mutants have a similar cell length distribution [7]. Using either ParZ-msfGFP or mCherry-ParB as a marker for *oriC* localization, we have previously shown that the control strains have ~10 copies of *oriC* per cell, but this number decreases to ~9 for Δ*parA*, 7–8 for *ΔparBS*, *ΔparZ*, *ΔparAZ*, and *Δsmc*, and ~6 for *ΔparAΔparBS* [7]. Additionally, Δ*parBS* and Δ*parAZ* both disrupt the even spacing of *oriC* in the polyploid cells, but Δ*parAZ* has the more pronounced effect that is similar to that of *ΔparZ* [7]. Importantly, the *ΔparAΔparBS* mutant has a much stronger defect in origin spacing than *ΔparA*, *ΔparBS*, *ΔparZ*, or *ΔparAZ*, lending support to the conclusion that ParA works with ParZ in a pathway separate from ParB/*parS* [7]. Although ParB/*parS* does not seem to interact with ParA in *B*. *burgdorferi*, our previous work has shown that ParB binds to *parS* and recruits Smc to the origin region [7], highlighting that the *parS*-ParB-Smc interactions in *B*. *burgdorferi* are similar to those demonstrated in other bacterial species [13, 14, 38, 39, 41]. Thus, in *B*. *burgdorferi*, the regular spacing of chromosome copies is controlled by two separate partitioning systems that involve the protein pairs ParA/ParZ and ParB/Smc [7].

**Fig 5:**
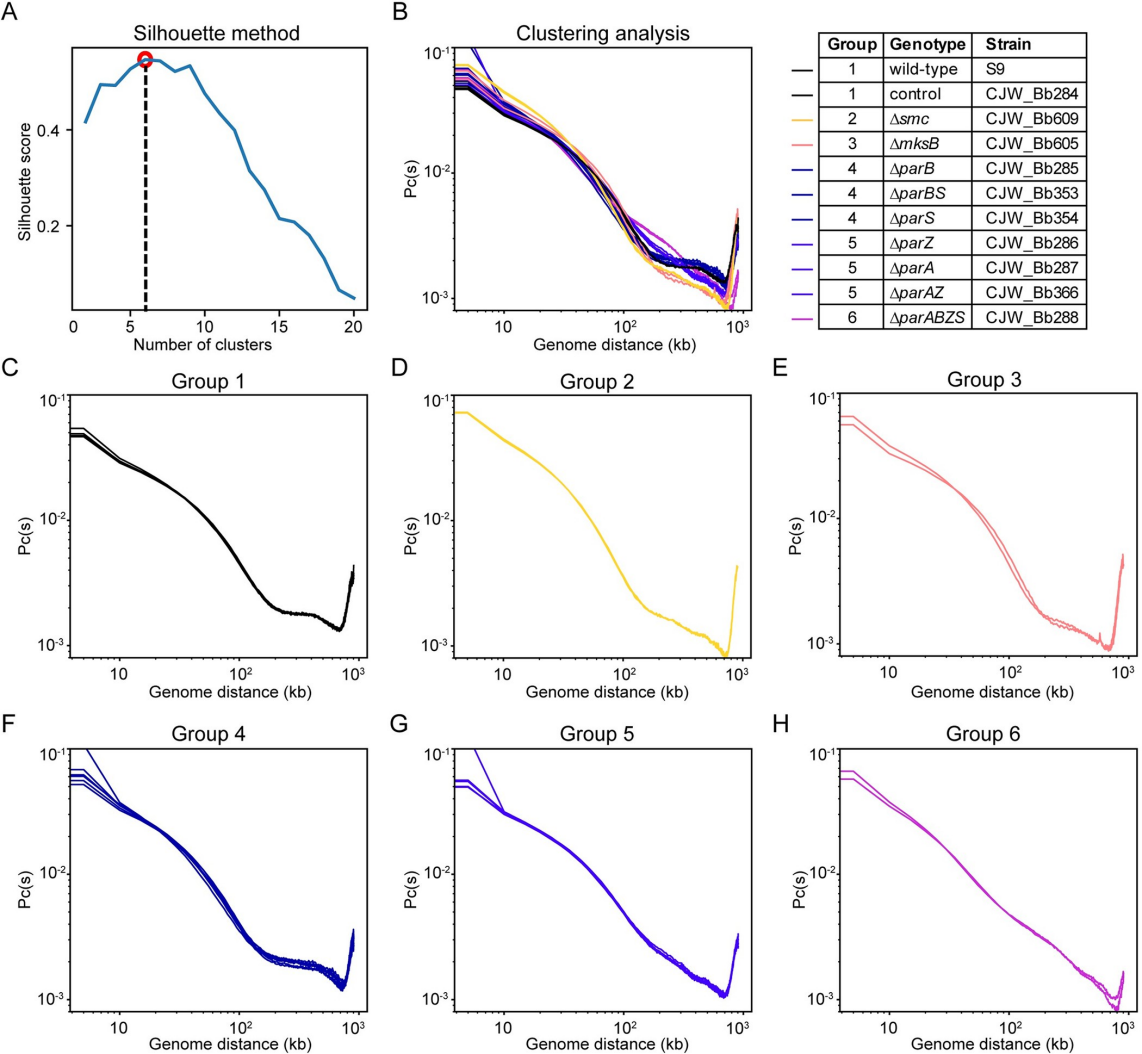
Clustering analysis of different mutants. **(A)** Determination of the optimal number of clusters of contact probability curves, Pc(s), for k-means clustering (see Materials and methods). Only intra-chromosomal interactions were used to calculate the Pc(s) curves. The number of clusters was determined by identifying the peak in Silhouette score. This analysis found six optimal groupings, which is indicated by the red circle and black dotted line. **(B)** Pc(s) curves of all the samples plotted in the same graph. Pc(s) curves show the average contact frequency between all pairs of loci on the chromosome separated by set distance (*s*). The x-axis indicates the genomic distance of separation in kb. The y-axis represents the averaged contact frequency. The curves were computed for intra-chromosomal interactions binned at 5 kb. Grouping result of the 11 strains was listed on the right. Two biological replicates of each strain were plotted. Individual Pc(s) curves can be found in **S4 Fig**. Principal Component Analysis (PCA) and T-distributed stochastic neighbor embedding (t-SNE) results can be found in **S5 and S6 Figs**, respectively. (**C-I**) Curves belonging to the same groups in (**B**) were plotted in different panels. Two biological replicates of each strain were plotted.

To understand the contribution of ParB/*parS*/Smc, ParA/ParZ, and additionally MksB to *B*. *burgdorferi* genome organization, we performed Hi-C on these mutants (**S1 Table**) and the control strain and compared the results with those of the WT. Hi-C experiments on every strain were done in two biological replicates which showed nearly identical results (**S3 Fig**). To compare the different mutants, we performed a clustering analysis using the contact probability curves of our 22 Hi-C samples **(S4 Fig)** so that mutants that had similar profiles of contact probabilities would be grouped together (**Fig 5**). Using the Silhouette method [54], we found that the mutants could be divided into six groups (**Fig 5A and 5B**) (see Materials and methods), which was largely consistent with Principal Component Analysis [54] (**S5 Fig**) and t-distributed stochastic neighbor embedding [54] (**S6 Fig**): group 1 included the WT and the control strain CJW_Bb284 (**Figs 5B, 5C and S7**); group 2 included *Δsmc* (**Fig 5B and 5D**); group 3 included *ΔmksB* (**Fig 5B and 5E**); group 4 included *ΔparB*, *ΔparS* and *ΔparBS* (**Fig 5B and 5F)**; group 5 included *ΔparA*, *ΔparZ* and *ΔparAZ* (**Fig 5B and 5G**); and group 6 included *ΔparAZBS* (**Fig 5B and 5H**).

This grouping analysis based on Hi-C results indicates that the control strain CJW_Bb284 behaves the same as its parental WT strain (**S7 Fig**); Smc and MksB have different effects on chromosome folding; ParB and *parS* work as a unit; ParA and ParZ work together; and ParB/*parS* and ParA/ParZ have additive effects because *ΔparAZBS* formed its own group. Therefore, the grouping of mutants based on Hi-C analysis here (**Fig 5B**) is largely consistent with our previous cytological characterization of these mutants [7]. This agreement shows the robustness of our assays.

### Smc and MksB mediate long-range interactions within the chromosome

**Fig 6:**
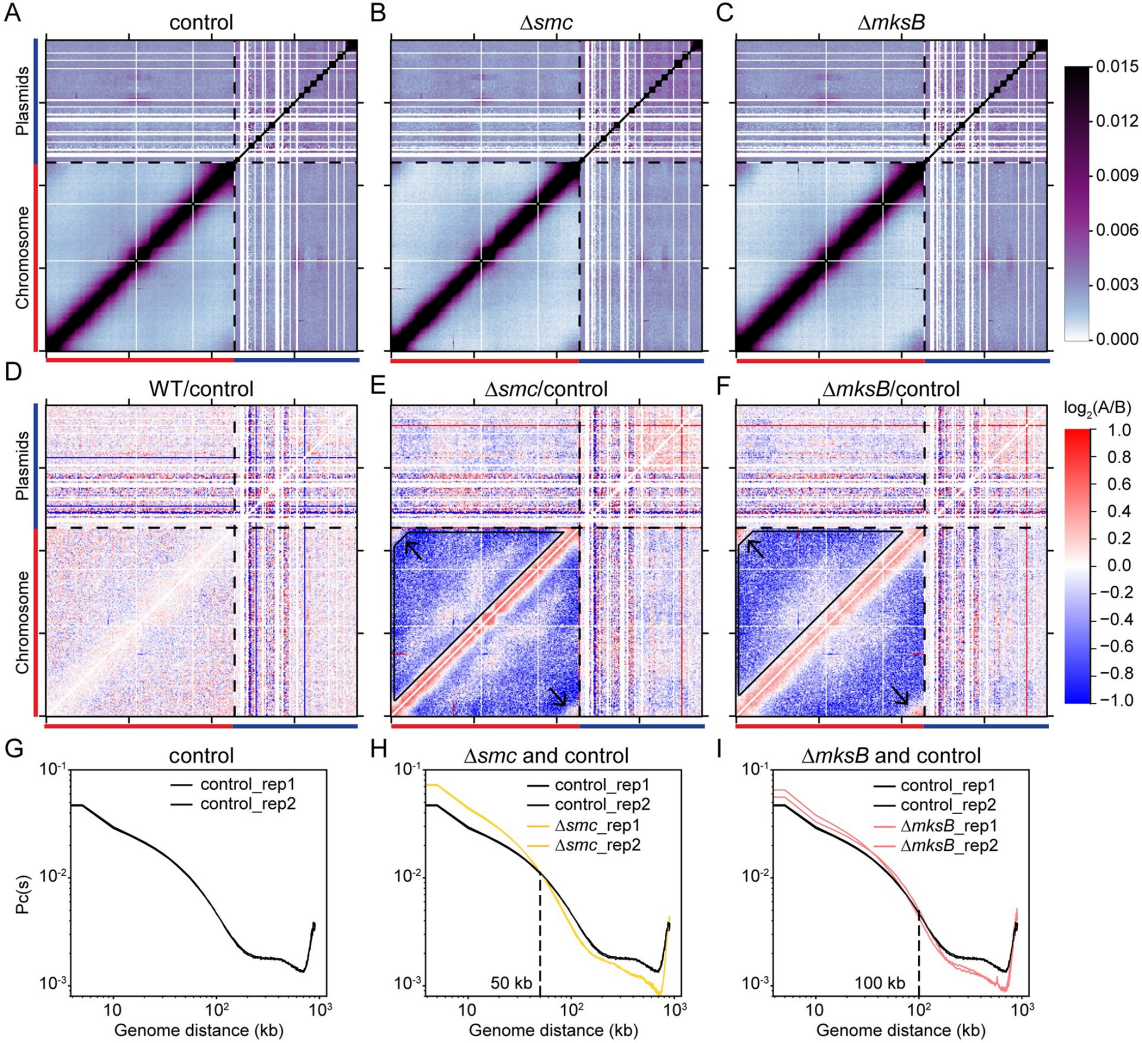
Smc and MksB mediate long-range DNA interactions. **(A-C)** Normalized Hi-C interaction maps of the control (CJW_Bb284), Δ*smc* (CJW_Bb609) and Δ*mksB* (CJW_Bb605) strains. Black dotted lines mark the boundary between the chromosome and the plasmids. The color scale depicting Hi-C interaction scores in arbitrary units is shown at the right. **(D-F)** Log_2_ ratio plots comparing different Hi-C matrices. Log_2_(matrix 1/matrix 2) was calculated and plotted in the heatmaps. Identities of matrix 1/ matrix 2 are shown at the top of each plot. The color scale is shown at the right of panel **(F)**. Black arrows point to *terCL-terCR* interactions. Black trapezoids indicate reduced interactions in the mutants. **(G-I)** Contact probability decay Pc(s) curves of indicated Hi-C matrices taken from **Fig 5B**. The intersection points of mutant and control curves are indicated by black dotted lines.

In our clustering analysis, the two biological replicates of *Δsmc* fell in one group (group 2) and replicates of *ΔmksB* fell into a separate group (group 3) (**Fig 5B, 5D and 5E)**. To understand how *Δsmc* and *ΔmksB* affect genome contacts, we analyzed the log_2_ ratios of the Hi-C maps between each mutant strain and the relevant control (**Fig 6A–6F**). We observed that both *Δsmc* and *ΔmksB* strains had decreased long-range DNA contact compared with the control (**Fig 6D–6F**, blue pixels in black trapezoid). Specifically, as seen on the Hi-C contact probability decay curves, in *Δsmc*, loci separated by ~50 kb or greater had decreased frequency of contacts compared with the control (**Fig 6G and 6H**), and in *ΔmksB*, loci separated by ~100 kb or greater had decreased frequency of contact compared with the control (**Fig 6G and 6I**, black dotted lines). These data indicate that both Smc and MksB promote long-range DNA contacts and that their effects are different enough to fall into different groups in our clustering analysis. We noted that *B*. *burgdorferi* is missing the ScpB subunit of the SMC complex, as well as the MksE and MksF subunits of the MksBEF complex. However, previous work showed that purified *B*. *subtilis* Smc protein (in the absence of ScpA and ScpB) is able to form DNA loops *in vitro* [55]. Our results suggest that in *B*. *burgdorferi*, the incomplete SMC/Mks complexes may form DNA loops. Alternatively, it is possible that *B*. *burgdorferi* uses unknown factors instead of ScpB and MksEF. Curiously, the absence of MksB, and to a lesser degree, the absence of Smc, enhanced the *terCL-terCR* interactions (**Fig 6E and 6F**, black arrows). Since this trend is the opposite of the overall reduction of long-range DNA interactions seen in the *Δsmc* and *ΔmksB* strains (**Fig 6E and 6F,** black trapezoids), these results suggest that MksB and Smc specifically reduce the contacts between the telomeres. In addition, when the data were normalized to remove intra-chromosomal interactions, we did not find evidence of MksB or Smc affecting plasmid-chromosome (**S8, S9 and S10 Figs)** or plasmid-plasmid interactions (**S11 and S12 Figs**), suggesting that these proteins act primarily within the chromosome and not between replicons. Finally, we do not know whether MksB and Smc affected the intra-replicon contacts within each plasmid because our 5-kb resolution was too low for the small sizes of the plasmids.

### Contribution of ParB/*parS* and ParA/ParZ to genome organization

**Fig 7:**
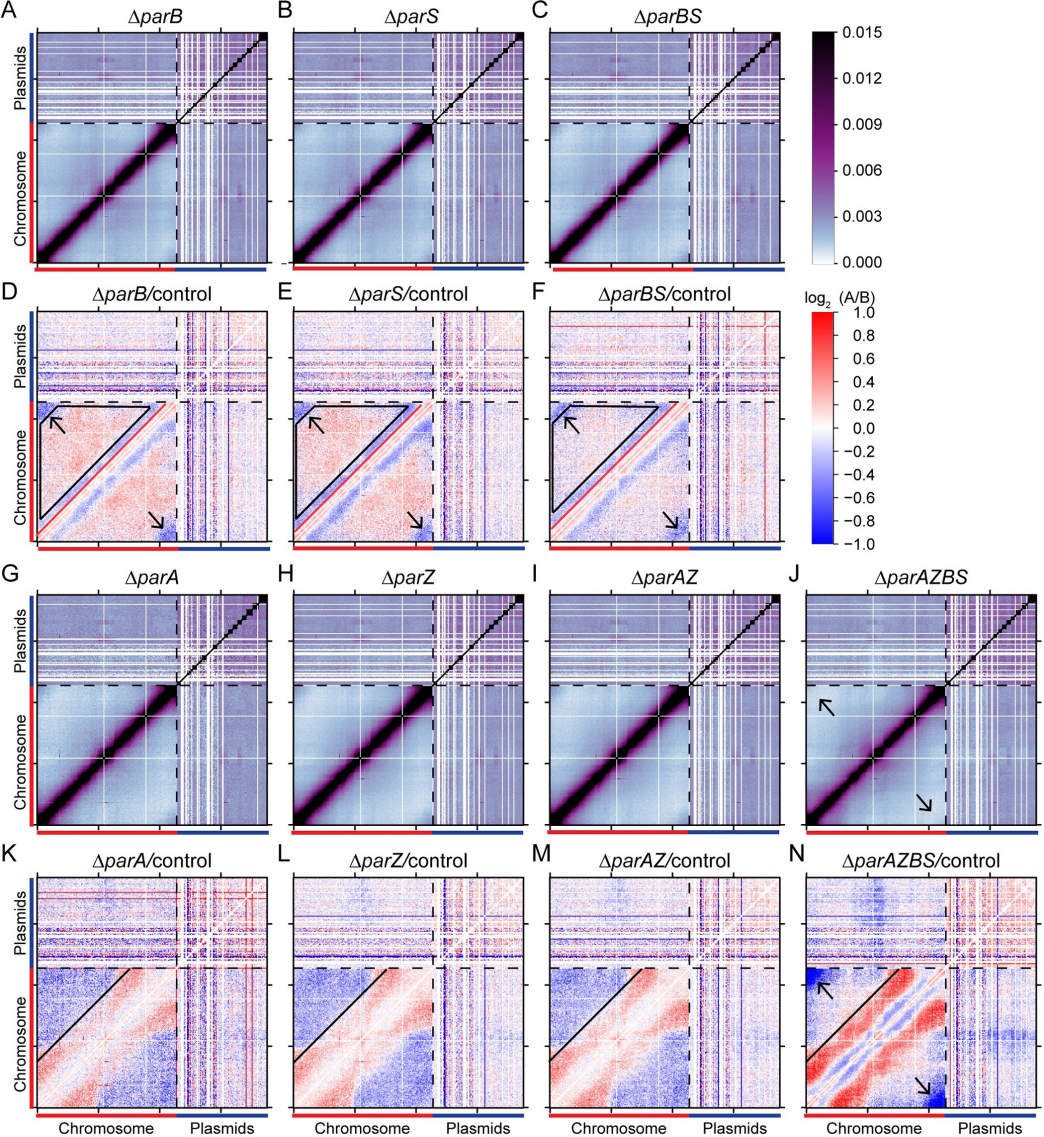
Disruption of the partition systems re-structures the genome. **(A-C)** Normalized Hi-C interaction maps of the Δ*parB* (CJW_Bb353), Δ*parS* (CJW_Bb354), and Δ*parBS* (CJW_Bb285) strains. Black dotted lines indicate the boundary between the chromosome and the plasmids. The color scale depicting Hi-C interaction scores in arbitrary units is shown at the right. **(D-F)** Log_2_ ratio plots comparing Δ*parB* (CJW_Bb353), Δ*parS* (CJW_Bb354), or Δ*parBS* (CJW_Bb285) with the control (CJW_Bb284) strain as indicated. Black arrows point to blue pixels of *terCL-terCR* interactions. Black trapezoids indicate area of red pixels. Red lines indicate the boundary between red and blue pixels. The color scale is shown at the right. **(G-J)** Normalized Hi-C interaction maps of the Δ*parA* (CJW_Bb366), Δ*parZ* (CJW_Bb286), Δ*parAZ* (CJW_Bb287) and Δ*parAZBS* (CJW_Bb288) strains. Black arrows indicate *terCL-terCR* interactions. **(K-N)** Log_2_ ratio plots comparing indicated strains. Solid black lines mark the boundaries between red and blue pixels. Black arrows indicate *terCL-terCR* interactions.

In the grouping analysis, Δ*parS*, Δ*parB* and Δ*parBS* fell in the same group (group 4) (**Fig 5B and 5F**), consistent with the previous finding that ParB and *parS* act as a unit [7]. Compared with the control, the absence of *parB* and/or *parS* caused similar changes to genome interactions (**Fig 7A–7F**): *terCL-terCR* interactions decreased (**Fig 7D–7F**, blue pixels indicated by black arrows); longer range (>150 kb) interactions within the chromosome increased (**Fig 7D–7F**, red pixels within black trapezoid); and short-range interactions (50–150 kb) decreased (**Fig 7D–7F**, blue pixels between black trapezoid and the red line). These trends are opposite to those observed in *Δsmc* or *ΔmksB* (**Fig 6E and 6F**). We postulate that the effect of ParB/*parS* on global chromosome conformation might be due to their effect on Smc distribution. Our previous work showed that ParB recruits Smc to the *oriC* region in *B*. *burgdorferi*, and the loss of *parBS* caused Smc localization to be more dispersed on nucleoid [7]. Thus, the increase of long-range interactions in the absence of ParB/*parS* suggests that non-specific loading of Smc to the chromosome outside of the *oriC* region (i.e. independent of ParB/*parS*) contributes greatly to long-range chromosome interactions.

Group 5 contains *ΔparA*, *ΔparZ*, *ΔparAZ* (**Figs 5B, 5G and 7G–7I**), consistent with the idea that ParA and ParZ work in the same pathway [7]. The absence of *parA* and/or *parZ* caused two major changes in chromosome folding: loci separated by 100 to 300 kb had increased interactions (**Fig 7K–7M**, red pixels below the black line) and loci separated by 300 kb or more had decreased interactions (**Fig 7K–7M**, blue pixels above the black line). Thus, ParA/ParZ acts to reduce mid-range (100–300 kb) and enhance long-range (>300 kb) DNA interactions on the chromosome. Since ParA/ParZ promotes chromosome segregation and spacing, we speculate that loss of ParA acting on DNA caused these changes in DNA interactions.

Finally, Δ*parAZBS*, which lacked both *parBS* and *parAZ*, formed its own group (group 6) (**Figs 5B, 5H, 7J and 7N**), consistent with its physiological and cytological behavior being the most severe in all of the mutants tested [7]. In Hi-C experiments, this mutant essentially exhibited an additive effect of *ΔparBS* (**Fig 7C and 7F**) and *ΔparAZ* (**Fig 7I and 7M**): decreased interactions below 150 kb (like in *ΔparBS*), increased mid-range (100–300 kb) interactions (as seen in *ΔparAZ*), and a complete loss of *terCL-terCR* interactions (**Fig 7J and 7N**, black arrows). These effects can be explained by the independent actions of ParB/*parS* and ParA/ParZ that we discussed above.

Overall, our Hi-C analyses of these mutants indicate that the perturbation of genome interactions is correlated with the previously observed cytological defects in chromosome positioning and segregation [7]. Interestingly, although DNA interactions within the chromosome were changed in cells missing *parBS* or *parAZ*, the interactions between replicons (plasmid-chromosome and plasmid-plasmid interactions) remained similar to the control (**S8–S12 Figs**). Only in Δ*parAZBS*, plasmid-chromosome interactions were reduced, and plasmid-plasmid interactions were more evened out. It is possible that in *ΔparAZBS*, the entanglement of different copies of chromosomes in the polyploid cells [7] affected the interactions between replicons.

## Discussion

In this study, we characterized the organization of the highly segmented genome of *B*. *burgdorferi* and the contribution of the chromosome partitioning proteins and Smc homologs to this organization. Even though *B*. *burgdorferi* expresses an Smc protein, we found that the chromosome does not have inter-arm interactions, which are observed in other Smc-carrying bacteria [34, 36, 38, 39, 41, 47, 48]. Nonetheless, Smc and the Smc-like MksB protein increase long-range DNA contacts possibly through DNA looping. Since *B*. *burgdorferi* lacks ScpB and MksEF thus cannot form complete SMC and Mks complexes, it is possible that the loop formation mechanism by the incomplete complexes is different from the loop-extrusion activity of the holocomplexes [55–59]. For instance, Smc or MksB alone might facilitate long-range loop formation by bridging only DNA segments that are already in proximity. Alternatively, just as ParA works with ParZ instead of ParB in *B*. *burgdorferi*, it is also possible that Smc and MksB recruit other factors instead of ScpB and MksEF in this organism.

The *B*. *burgdorferi* strain used in this study contains 18 plasmids, which showed differential interactions with the chromosome. Namely, plasmids lp17, lp21, lp25, and lp28-3 displayed higher frequency of contact with the chromosome especially at the *oriC* region (**Figs 3A and S8**). This pattern was highly reproducible in different mutants (**S8–S10 Figs**), suggesting that these plasmid-chromosome contacts are real, specific interactions that might be mediated by unknown protein factors. We did not detect specific plasmid-*oriC* colocalization in our previous imaging-based analysis [7]. This is likely because these interactions are transient, and such weak but reproducible interactions are more easily captured in Hi-C experiments where millions of cells are averaged than in microscopy experiments where fewer cells are analyzed.

What are the molecular mechanism and biological function of these plasmid-chromosome interactions? In *A*. *tumefaciens*, the secondary replicons cluster with the primary replicon at their origin regions through the interactions between ParB homologs [41, 42], which prevents the loss of the secondary replicons [42]. In *B*. *burgdorferi*, we note that these interactions did not require ParB/*parS* or ParA/ParZ (**S8–S10 Figs**), suggesting that the molecular mechanism for these interactions is different from the centromeric clustering observed in *A*. *tumefaciens*. Although it is still possible that the four plasmids that interact with the chromosome may “piggyback” the chromosome to facilitate their own segregation and maintenance, it is also possible that these plasmid-chromosome interactions have functions unrelated to plasmid segregation. Indeed, 14 out of 18 plasmids did not interact with the chromosome origin, indicating that *B*. *burgdorferi* plasmids segregate largely independently from the chromosome. Notably, *B*. *burgdorferi* is polyploid with unequal copy number for each replicon [7] while *A*. *tumefaciens* newborn cells are haploid [41]. We postulate that the difference in ploidy might be one underlying factor accounting for the difference in organizing strategies between these two species. Our findings suggest that different species might take diverse strategies to organize and maintain segmented genomes.

We found that the interactions between the plasmids on average are more frequent than plasmid-chromosome interactions and long-range intra-chromosomal interactions (**Figs 1B and 2**). Interestingly, all seven circular cp32 plasmids interacted more frequently with one another (**Fig 4B and 4C**); the remaining 11 plasmids, including the circular cp26 plasmid and the ten linear plasmids, preferentially interacted with one another, though to a lesser degree (**Fig 4C**). These groupings cannot be simply explained by plasmid size, topology, or copy number (**Figs 1A and 4D**). In addition, all the *B*. *burgdorferi* plasmids are thought to use members of the PF32, PF49, PF50 and PF57/62 gene clusters for replication and partitioning [4, 60–62]: PF32 belongs to the ParA protein family, PF50 and PF57/62 are homologs of replication initiator proteins, while PF49 likely serves as a ParB-like centromeric protein [63]. Therefore, their replication and partitioning systems cannot explain the grouping of the plasmids, either. Curiously, cp32 plasmids resemble the genomes of certain tailed bacteriophages [5, 64–66] and cp32 DNA was found to be packaged in bacteriophage particles isolated from *B*. *burgdorferi* cultures [67]. Thus, it is conceivable that the grouping of cp32 plasmids might be related to the process of bacteriophage assembly, although the phage proteins are expressed at minimal level without induction [5, 68]. The exact mechanism for the preferential interactions between plasmids remains to be explored.

Unlike in other bacteria studied to date, in *B*. *burgdorferi*, there are two partitioning systems, ParA/ParZ and ParB/*parS*, which co-regulate the spacing of the *oriC* copies in the cell. ParA/ParZ plays a more important role than ParB/*parS*. While removing ParB/*parS* only causes very mild defects in *oriC* spacing in the presence of ParA/ParZ, deleting both *parA* and *parBS* further disrupts the spacing pattern [7]. By Hi-C, we observed a similar trend in genome reorganization in these mutants: removing *parAZ* caused a significant increase of the medium-range (100–300 kb) interactions but double deletion of *parAZ* and *parBS* led to an additive increase in these interactions. Thus, the segregation defect is correlated with increased mid-range genome interactions. The causal relationship between chromosome segregation and genome folding is unclear and remains to be examined. We speculate that the tension exerted through the partitioning system leads to the change in DNA folding over the length of the chromosome, which is a decrease of DNA interactions in the 100–300 kb range observed here.

Despite the absence of inter-arm interactions on the chromosome, the two ends of the linear chromosome, *terCL* and *terCR*, displayed a high contact frequency, which required ParA/ParZ and ParB/*parS*. The contribution of ParA/ParZ and ParB/*parS* to *terCL*-*terCR* interactions might be through different mechanisms. ParA/ParZ is required for the spacing of *oriC* copies [7]. Thus, it is possible that mis-positioning of chromosome copies reduces the frequency of *terCL-terCR* contacts. For ParB/*parS*, although it does not contribute much to the spacing of chromosome copies [7], it recruits Smc to the origin. Since Smc reduced *terCL-terCR* contacts (**Fig 5F**), it is possible that ParB-mediated recruitment of Smc to the *oriC*-proximal *parS* site and away from chromosome arms lifts Smc’s inhibitory role in *terCL-terCR* interactions.

Altogether, our study identified intra-chromosomal, plasmid-chromosome, and plasmid-plasmid interactions of the most segmented bacterial genome known to date. We explored the contribution of SMC-family proteins and two partitioning systems to the folding and interactions of the genome. Although the exact mechanism for replicon interactions remains to be investigated, our study represents one step forward in the understanding of multipartite genome architecture and maintenance.

## Materials and methods

### General methods

The *B*. *burgdorferi* strains used in this study are listed in **S1 Table**. Cells were maintained in exponential growth in complete Barbour-Stoenner-Kelly (BSK)-II liquid medium at 34°C in a humidified incubator and under 5% CO2 atmosphere [69, 70]. Complete BSK-II medium contained 50 g/L bovine serum albumin (Millipore, Cat. 810036), 9.7 g/L CMRL-1066 (US Biological, Cat. C5900-01), 5 g/L Neopeptone (Difco, Cat. 211681), 2 g/L Yeastolate (Difco, Cat. 255772), 6 g/L HEPES (Millipore, Cat. 391338), 5 g/L glucose (Sigma-Aldrich, Cat. G7021), 2.2 g/L sodium bicarbonate (Sigma-Aldrich, Cat. S5761), 0.8 g/L sodium pyruvate (Sigma-Aldrich, Cat. P5280), 0.7 g/L sodium citrate (Fisher Scientific, Cat. BP327), 0.4 g/L N-acetylglucosamine (Sigma-Aldrich, Cat. A3286), 60 mL/L heat-inactivated rabbit serum (Gibco, Cat.16120), and had a final pH of 7.60. When noted, the following antibiotics were used: gentamicin at 40 μg/mL, streptomycin at 100 μg/mL, and kanamycin at 200 μg/mL [71–73]. Lists of plasmids, oligonucleotides and next-generation-sequencing samples can be found in **S2–S4 Tables**.

### Growing cells for Hi-C

For Hi-C biological replicates, pairs of 100 mL cultures of each strain were inoculated and grown for two or three days. The cultures were fixed by adding 37 mL 37% formaldehyde (Sigma-Aldrich, Cat. F8775) which resulted in 10% final concentration. This formaldehyde concentration was chosen because the BSK-II medium used in this study was rich in primary amines (see General methods above) which reacted with formaldehyde. 10% formaldehyde gave us highly reproducible Hi-C results without signs of over-crosslinking such as inefficient lysis or digestion. For crosslinking, the cultures were rocked at room temperature for 30 min. Formaldehyde was quenched using 7 mL 2.5 M glycine at room temperature for 5 min with rocking. The samples were chilled on ice for 10 min, then pelleted at 4°C and 4,300 x g for 30 min in an Allegra X-14R centrifuge (Beckman Coulter) equipped with a swinging bucket SX4750 rotor. The cell pellet was resuspended in 1 mL ice-cold HN buffer (50 mM NaCl, 10 mM HEPES, pH 8.0) [74], then pelleted at 4°C and 10,000 x g for 10 min. The pellet was resuspended in 400 μL cold HN buffer, and 100 μL aliquots were frozen in a dry ice ethanol bath then stored at below -80°C.

### Hi-C

The detailed Hi-C procedure for *B*. *burgdorferi* was adapted from previously described protocols for *B*. *subtilis* [34] and *A*. *tumefaciens* [41]. Briefly, 5x10^8^ *B*. *burgdorferi* cells were used for each Hi-C reaction. Cells were lysed using Ready-Lyse Lysozyme (Epicentre, R1802M) in TE for 60 min, followed by 0.5% SDS treatment for 30 min. Solubilized chromatin was digested with DpnII for 2 hours at 37°C. The digested chromatin ends were repaired with Klenow and Biotin-14-dATP, dGTP, dCTP, dTTP. The repaired products were ligated in dilute reactions by T4 DNA ligase at 16°C overnight (about 20 hrs). Ligation products were incubated at 65°C overnight to reverse crosslinking in the presence of EDTA, 0.5% SDS and proteinase K. The DNA was then extracted twice with phenol/chloroform/isoamylalcohol (25:24:1) (PCI), precipitated with ethanol, and resuspended in 40 μL 0.1XTE buffer. Biotin at non-ligated ends was removed using T4 polymerase (4 hrs at 20°C) followed by extraction with PCI. The DNA was then resuspended in 105 μL ddH_2_O and sheared by sonication for 12 min with 20% amplitude using a Qsonica Q800R2 water bath sonicator. The sheared DNA was used for library preparation with the NEBNext UltraII kit (E7645) following the manufacturer’s instructions for end repair, adapter ligation, and size selection. Biotinylated DNA fragments were purified using 5 μL streptavidin beads (Invitrogen 65-001) following the manufacturer’s instructions. All DNA-bound beads were used for PCR in a 50 μL reaction for 14 cycles. PCR products were purified using Ampure beads (Beckman, A63881) and sequenced at the Indiana University Center for Genomics and Bioinformatics using a NextSeq 500 sequencer.

### Hi-C analysis

Paired-end sequencing reads were mapped to the genome file of *B*. *burgdorferi* B31 (NCBI Reference Sequence GCA_000008685.2 ASM868v2) using the default setting with MAPQ30 filter of Distiller (https://github.com/open2c/distiller-nf). Plasmids are arranged in this order: cp26, cp32-1, cp32-3, cp32-4, cp32-6, cp32-7, cp32-8, cp32-9, lp17, lp21, lp25, lp28-1, lp28-2, lp28-3, lp28-4, lp36, lp38 and lp54. Plasmids cp9, lp5 and lp56 are absent from our strain.

The mapped Hi-C contact frequencies were stored in multi-resolution cooler files [75] and the Hi-C matrices were balanced using the iterative correction and eigenvector decomposition method [49]. The iterative correction method is a standard way to balance the Hi-C map such that the rows and columns sum to a constant value (typically 1), which helps to correct for biases in genomic coverage, for example some genomic regions might be easier to amplify than other regions. The iterative correction process can be roughly summarized as follows. Each individual value within a row is divided by the sum of values for that row to achieve a sum of 1 for every row. However, this normalization of the rows breaks the required symmetry of the Hi-C matrix. Therefore, row normalization is followed by column normalization in which each individual value in a column is divided by the resulting sum of values for that column, which subsequently "unbalances" the rows and the row sum is no longer 1. As such, the process is iteratively repeated until the row and column sums converge to 1 within a pre-defined error tolerance for which we used the default value of 10^−5^. This results in a balanced Hi-C matrix in which genomic coverage biases are minimized. We described the process starting with normalization of rows followed by columns. However, the procedure could equally have been applied by starting with columns instead of rows since the Hi-C matrix is symmetric about the primary diagonal. Unless otherwise specified, all Hi-C plots and downstream analyses were performed with this iterative correction. For the renormalization of plasmid-chromosome and plasmid-plasmid interactions (**Figs 3C, 4C, S9 and S12**), the same procedure of iterative correction was used.

Plots were generated with R or Python 3.8.15 using Matplotlib 3.6.2 [76]. Data were retrieved for plotting at 5-kb resolution. Pc(s) curves show the averaged contact frequency between all pairs of loci on the chromosome separated by set distance (s). The x-axis indicates the genomic distance of separation in kb. The y-axis represents the averaged contact frequency in a logarithmic scale. The curves were computed for data binned at 5 kb. For the log_2_ ratio plots, the Hi-C matrix of each mutant was divided by the matrix of the control. Then, log_2_(mutant/control) was calculated and plotted in a heatmap using R.

### Indicating highly transcribed genes on a Hi-C map

The RNA-seq data of the *B*. *burgdorferi* B31-S9 strain growing in culture from a recent published study (SRR22149536) [46] were mapped to WT *B*. *burgdorferi* B31 genome (NCBI GCA_000008685.2_ASM868v2) using CLC Genomics Workbench (QIAGEN) as previously described [7]. RNA-seq analysis was performed using the default setting of the built-in package of CLC Genomics Workbench. Genes were ranked by transcripts per kilobase per million reads (TPM). For the top 50 highly transcribed genes, the first nucleotide of each gene was indicated with fine dotted lines and plotted on to the Hi-C map using R (**S1B Fig**).

### Clustering of strains based on Hi-C data

Clustering of strains based on the contact probability curves was done using the scikit-learn 1.1.3 k-means algorithm [54]. The optimal number of clusters was determined using the maximum of the Silhouette score. The Silhouette score, *s(i)* is a metric that determines, for some collection of objects {i}, how well each individual object, *i*, matches the clustering at hand [77]. In our case, the collection of objects were the log-transformed contact frequency Pc(s) curves, which were computed as the average value of the contact frequency of pairs of loci separated by a fixed genomic distance. Average Silhouette scores were computed for data clustered using k-means with varying the number of clusters ranging from 2 to 21. We found that the number of clusters that maximized the average Silhouette score was six, suggesting that six is the optimal number of clusters in the data.

To better visualize the results of the k-means clustering and Silhouette method of identifying the optimal number of clusters, we visualized the data clusters using two different methods: Principal Component Analysis (PCA) and t-distributed stochastic neighbor embedding (t-SNE). PCA was performed using scikit learn 1.2.2 (sklearn.decomposition.PCA) [54] on the log-transformed Pc(s) curves (computed for the chromosome only, ignoring plasmids) for each of the 22 different Hi-C maps (11 strains, with 2 biological replicates each). To visualize how the data clusters together, we projected the Pc(s) curve values from each experiment onto the first two principal components, which explained approximately 85% of the total data variance (48% for component 1 and 37% for component 2). t-SNE was performed using scikit learn 1.2.2 (sklearn.maniforld.TSNE) [54] on the same input data used for the PCA (see above). We ran the t-SNE using the following parameters: n_components = 2, perplexity = 5, init = "random", n_iter = 2000, random_state = 0. The results were subsequently plotted in a two-dimensional graph, and the points of the scatter plot were labelled using the group classifications from application of the k-means clustering in Fig 5B.

### Simulating plasmid-plasmid interaction frequencies based on plasmid sizes and copy numbers

Plasmid-plasmid interaction frequencies were simulated assuming random collisions. We accounted for either plasmid copy numbers alone, or in combination with information on the plasmid lengths (**Fig 1A**). Plasmid copy numbers were previously determined using marker frequency analysis [7], which yielded values ranging between 0.5 and 1.4 relative to the *oriC* (see **Fig 1A**). Plasmid sizes ranged from 17–54 kb [3] (see **Fig 1A**), which covered 3–11 of 5-kb bins.

For the simulated plasmid-plasmid contact map using both the copy numbers and plasmid lengths (**S2A Fig**), we first multiplied the average plasmid copy number by the plasmid lengths in numbers of 5-kb bins and rounded the resulting number to the nearest integer, *n*_*p*_ for each plasmid *p*. The values of *n*_*p*_ ranged between 2 and 14, and the total sum over all the plasmids, *p*, was N = ∑*_p_n_p_* = 80. The simulated plasmid-plasmid “contact frequency” matrix was computed using the probability of randomly drawing a given pair of plasmids. The probability for drawing a plasmid, *p*, is *n*_*p*_/N. The resulting probability matrix from this calculation can be seen in **S2A Fig** (top panel). To best compare the simulated plasmid-plasmid contact probability map with the experimental Hi-C data, we applied the iterative correction procedure [49] to this map. The resulting matrix is shown both with the same scale bar as the experimental Hi-C map (**S2A Fig**, middle panel) and with a very fine color scale (**S2A Fig**, bottom panel). We note that the iterative correction scheme tends to minimize the effects of copy number variation from one genome segment to another and this is why the simulated plasmid-plasmid contact map looks largely uniform when plotted with the same dynamic range as experimental data (**Figs 4D and S2** middle panel).

The simulated plasmid-plasmid contact map computed using only copy numbers was made in a similar fashion (**S2B Fig**). For this method, instead of multiplying copy number by the length of the plasmid, a fixed integer number was used (in our case, 10) to convert the relative ratios into integer numbers. The method of computation was the same as that described above.

We made two assumptions for this simulation: 1) plasmids constitute independent units of interaction, and 2) plasmids are “well mixed”. The “independence of contact” assumption implies that there are no restrictions on how many DNA segments may be simultaneously in contact with one another and the identity of the DNA segments in contact does not matter. The “well mixed” assumption stipulates that independent DNA segments interact with equal probability with other DNA segments. Together, these assumptions allow us to compute the plasmid-plasmid interaction frequencies while safely ignoring other types of contacts such as plasmid-chromosome and chromosome-chromosome contacts. Our simulation does not consider the cytoplasmic volume.

### Plasmid construction

Plasmid pΔmksB(gent) was generated in the following manner: (i) nucleotides 874996 through 876527 of the B31 chromosome were PCR-amplified with primers NT968 and NT969; (ii) the gentamicin cassette of pKIGent_parSP1_phoU [7] was PCR-amplified with primers NT970 and NT971; (iii) nucleotides 879168 through 880691 of the B31 chromosome were PCR-amplified with primers NT972 and NT973; (iv) the suicide vector backbone of pΔparA(kan) [7] was PCR-amplified with primers NT974 and NT975; and (v) the four PCR fragments listed above were digested with DpnI (New England Biolabs), gel-purified, and subjected to Gibson assembly [78] using New England Biolabs’ platform. The assembled plasmid was introduced into *Escherichia coli* strain NEB 5-alpha (New England Biolabs) by heat shocking. The resulting strain (CJW7512) was grown at 30°C on LB plates or in Super Broth liquid medium with shaking, while 15 μg/mL gentamicin was used for selection.

### Strain construction

To generate strain CJW_Bb605, 75 μg of plasmid pΔmksB(gent) were digested with ApaLI (New England Biolabs) in a 500 μL reaction volume for 4 hours. The DNA was then ethanol precipitated [79], dried, and resuspended in 25 μL sterile water. The resulting DNA suspension was then electroporated at 2.5 kV, 25 μF, 200 Ω, in 2 mm-gap cuvette [80, 81] into 100 μL of electrocompetent cells made [82] using *B*. *burgdorferi* strain S9. The electroporated bacteria were transferred immediately to 6 mL BSK-II medium and allowed to recover overnight at 34°C. The next day, a fraction of the culture was embedded in 25 mL of semisolid BSK-agarose medium containing gentamicin per 10-cm round Petri dish, as previously described [83]. The semisolid BSK-agarose mix was made by mixing 2 volumes of 1.7% agarose in water, sterilized by autoclaving, then melted and pre-equilibrated at 55°C, with 3 volumes of BSK-1.5 medium, which was also equilibrated at 55°C for at most 5 minutes. BSK-1.5 contained 69.4 g/L bovine serum albumin, 12.7 g/L CMRL-1066, 6.9 g/L Neopeptone, 3.5 g/L Yeastolate, 8.3 g/L HEPES, 6.9 g/L glucose, 6.4 g/L sodium bicarbonate, 1.1 g/L sodium pyruvate, 1.0 g/L sodium citrate, 0.6 g/L N-acetylglucosamine, and 40 mL/L heat-inactivated rabbit serum, and had a final pH of 7.50. After 10 days of growth in the BSK-agarose semisolid matrix, an individual colony was expanded in liquid culture and confirmed by PCR to have undergone correct double crossover homologous recombination of the suicide vector, thus yielding strain CJW_Bb605. This strain was also confirmed by multiplex PCR [84] to contain all endogenous plasmids contained by its parent.

Requests for strains, plasmids, resources, reagents should be directed to and will be fulfilled by the corresponding authors with appropriate Material Transfer Agreements.

## Supporting information

S1 Fig Hi-C interaction map of *B*. *burgdorferi* strain S9 shown in a different color scale. **(A)** To better show the intra-chromosomal interactions in **Fig 1B**, the normalized Hi-C interaction map is shown in a different color scale. Black arrows point to a few examples of strong CID boundaries that overlap with highly transcribed genes shown in (**B**). The color scale depicting Hi-C interaction scores in arbitrary units is shown at the right. **(B)** The positions of the top 50 highly transcribed chromosomal genes found by RNA-seq [46] are indicated using fine black dotted lines. A recent study [46] published RNA-seq data of the *B*. *burgdorferi* B31-S9 strain grown in culture. We mapped the data to the *B*. *burgdorferi* B31 genome, calculated the number of transcripts per kilobase per million reads for each gene, and indicated the top 50 highly transcribed genes on the Hi-C map. Although the growth condition in our study was different from the RNA-seq study [46], strong CIDs boundaries (black arrows in **A**) largely overlap with highly transcribed genes.
(TIF)

S2 Fig Simulated plasmid-plasmid interaction frequencies. The contact probability between plasmids was simulated under the assumptions that plasmids are randomly interacting, independent of one another, and are “well mixed” within the cytoplasm (see Materials and methods). The calculation was performed accounting for plasmid copy numbers and plasmid lengths together (**A**) or only plasmid copy numbers (**B**). Top panels, the raw contact frequency expected between plasmids without normalization. Middle panels, the simulated contact frequency after normalization using iterative correction. Bottom panels, the same as middle panels, but shown with a much finer color scale. The color scales depicting contact frequencies in arbitrary units are shown at the right. We note that there is residual resemblance between bottom and top panels, and in the bottom panel, the row or column sums do not appear to be the same. This is because the iterative correction procedure stops when the row and column sums approach 1 within a pre-defined error tolerance (see Materials and methods), but not exactly at 1.
(TIF)

S3 Fig Hi-C samples used in this study. The normalized Hi-C interaction maps of all 22 experiments done for this study. The color scale depicting Hi-C interaction scores is shown at the bottom right.
(TIF)

S4 Fig Individual Pc(s) curves of all the samples analyzed in this study. Pc(s) curves of all 22 Hi-C experiments done in this study. The x-axis indicates genomic distance while the y-axis shows averaged contact frequency. Only intra-chromosomal interactions were used to calculate the Pc(s) curves.
(TIF)

S5 Fig Principal Component Analysis (PCA) with groups from k-means clustering results. To better visualize the results of the k-means clustering generated by the Silhouette method, we performed Principal Component Analysis (PCA) and labeled the clustering results (see Materials and methods). The plots with up to six clusters gave nicely visually segregated groups. Beyond six, the two-dimensional projections from PCA showed poor segregation of the data points, and biological replicates were separated to different groups.
(TIF)

S6 Fig T-distributed stochastic neighbor embedding (t-SNE) with groups from k-means clustering results. To better visualize the results of the k-means clustering generated by the Silhouette method, we performed t-distributed stochastic neighbor embedding (t-SNE) and labeled the clustering results (see Materials and methods). Similar to PCA, the plots with up to six clusters gave nicely visually segregated groups. Beyond six, the two-dimensional projections from t-SNE showed poor segregation of the data points, and biological replicates were separated to different groups.
(TIF)

S7 Fig Comparison of WT and control strains. **(A-B)** Normalized Hi-C interaction maps of *B*. *burgdorferi* strains S9 (WT) and the control strain CJW_Bb284. Two biological replicates of each strain (rep1 and rep2) are shown. The color scale depicting Hi-C interaction scores in arbitrary units is shown at the right. **(C)** Pc(s) curves of the four samples. Pc(s) curves show the averaged contact frequency between all pairs of loci on the chromosome separated by set distance (s). The x-axis indicates the genomic distance of separation in kb. The y-axis represents the averaged contact frequency. The curves were computed for data binned at 5 kb. Only intra-chromosomal interactions were used to calculate the Pc(s) curves. **(D-F)** Log_2_ ratio plots comparing different Hi-C matrices. Log_2_(matrix 1/matrix 2) was calculated and plotted in the heatmaps. The identities of matrix 1/matrix 2 are shown at the top of each plot. The color scale is shown at the right of panel **(F)**.
(TIF)

S8 Fig Plasmid-chromosome interactions in different mutants. Calculated plasmid-chromosome interaction frequencies are shown. The x-axis shows chromosome location in kb. The y-axis specifies the different plasmids analyzed. The color indicates the contact frequency between each plasmid and chromosome locus. Each graph plots the mean value of the two biological replicates shown in **S3 Fig**. Data are binned at 5-kb resolution. The data were normalized including all the interactions in the genome (i.e. intra-chromosomal, plasmid-chromosome and plasmid-plasmid interactions).
(TIF)

S9 Fig Renormalized plasmid-chromosome interactions in different mutants. Plasmid-chromosome interactions from **S8 Fig** were renormalized using iterative correction to remove the influence of intra-chromosomal and plasmid-plasmid interactions (see Materials and methods). The data were normalized such that each row had the same total score, and each column had the same total score.
(TIF)

S10 Fig Plasmid-chromosome interactions in different mutants organized by plasmids. Calculated plasmid-chromosome interaction frequencies are shown. The x-axis shows the chromosome location in kb. The y-axis specifies the different mutants. The color indicates the contact frequency between each plasmid and chromosome locus. Each graph plots the mean value of the two biological replicates shown in **S3 Fig**. Data are binned at 5-kb resolution.
(TIF)

S11 Fig Plasmid-plasmid interactions in different mutants. Calculated plasmid-plasmid contact frequencies in different strains. The x- and y-axes indicate the plasmids analyzed. The color shows the computed contact frequency. Each graph plots the mean of the two biological replicates shown in **S3 Fig**. The data were normalized including all the interactions in the genome (i.e. intra-chromosomal, plasmid-chromosome and plasmid-plasmid interactions).
(TIF)

S12 Fig Renormalized plasmid-plasmid interactions in different mutants. Plasmid-plasmid contact frequencies from **S11 Fig** were renormalized without plasmid-chromosome interactions. The data were normalized such that each row had the same total score, and each column had the same total score.
(TIF)

S1 Table Bacterial strains used in this study. (DOCX)

S2 Table Plasmids used in this study. (DOCX)

S3 Table Oligonucleotides used in this study. (DOCX)

S4 Table Next-generation-sequencing samples used in this study. (DOCX)
