# Supplementary figures and images for: Organization and replicon interactions within the highly segmented genome of Borrelia burgdorferi
Source: PLoS Genet. 2023 Jul 26;19(7):e1010857. doi: 10.1371/journal.pgen.1010857 (PMC10406323; doi:10.1371/journal.pgen.1010857)

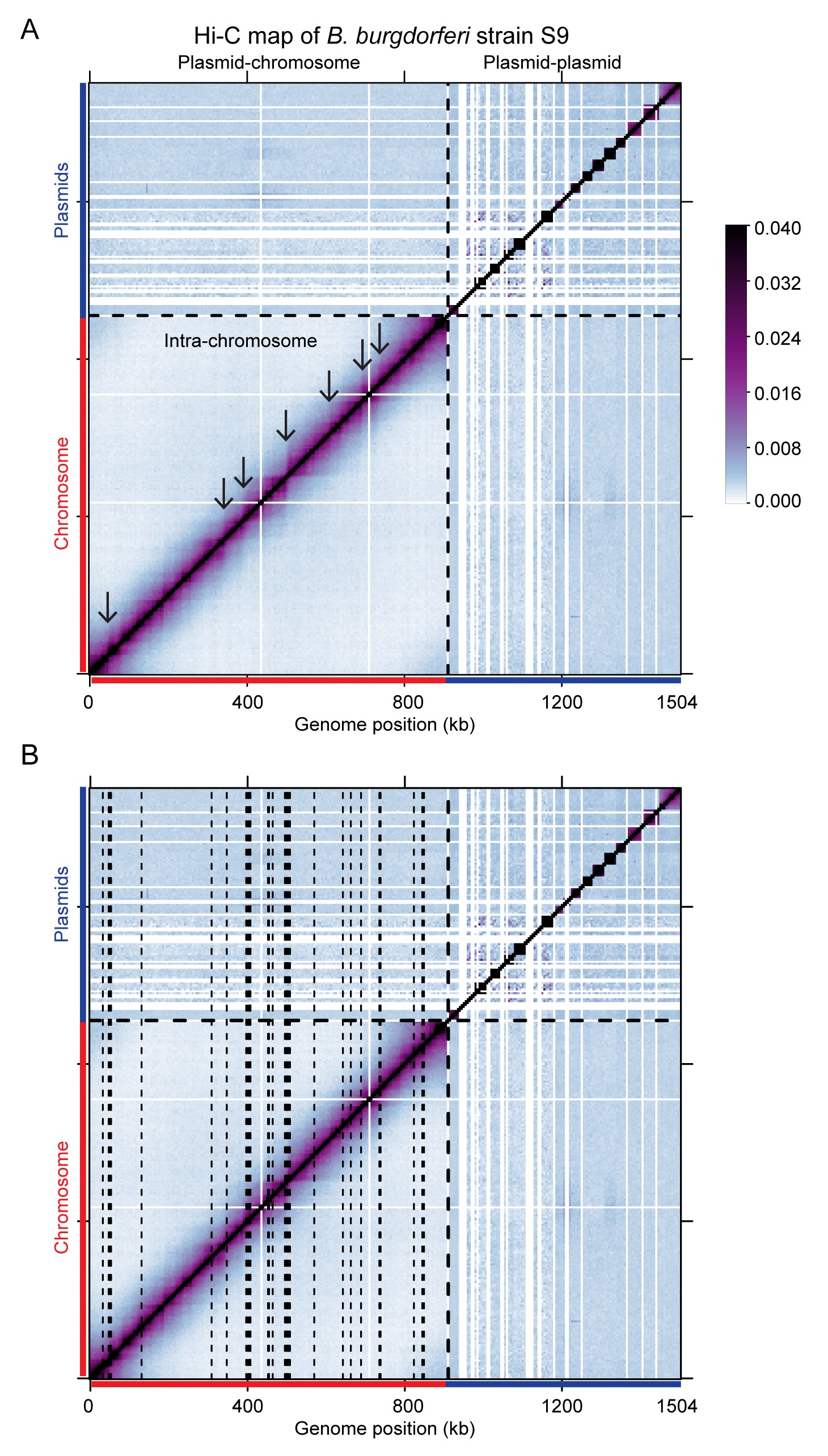

Supplement: S1 Fig — (A) To better show the intra-chromosomal interactions in Fig 1B, the normalized Hi-C interaction map is shown in a different color scale. Black arrows point to a few examples of strong CID boundaries that overlap with highly transcribed genes shown in (B). The color scale depicting Hi-C interaction scores in arbitrary units is shown at the right. (B) The positions of the top 50 highly transcribed chromosomal genes found by RNA-seq [46] are indicated using fine black dotted lines. A recent study [46] published RNA-seq data of the B. burgdorferi B31-S9 strain grown in culture. We mapped the data to the B. burgdorferi B31 genome, calculated the number of transcripts per kilobase per million reads for each gene, and indicated the top 50 highly transcribed genes on the Hi-C map. Although the growth condition in our study was different from the RNA-seq study [46], strong CIDs boundaries (black arrows in A) largely overlap with highly transcribed genes. (TIF) [file pgen.1010857.s001.tif]

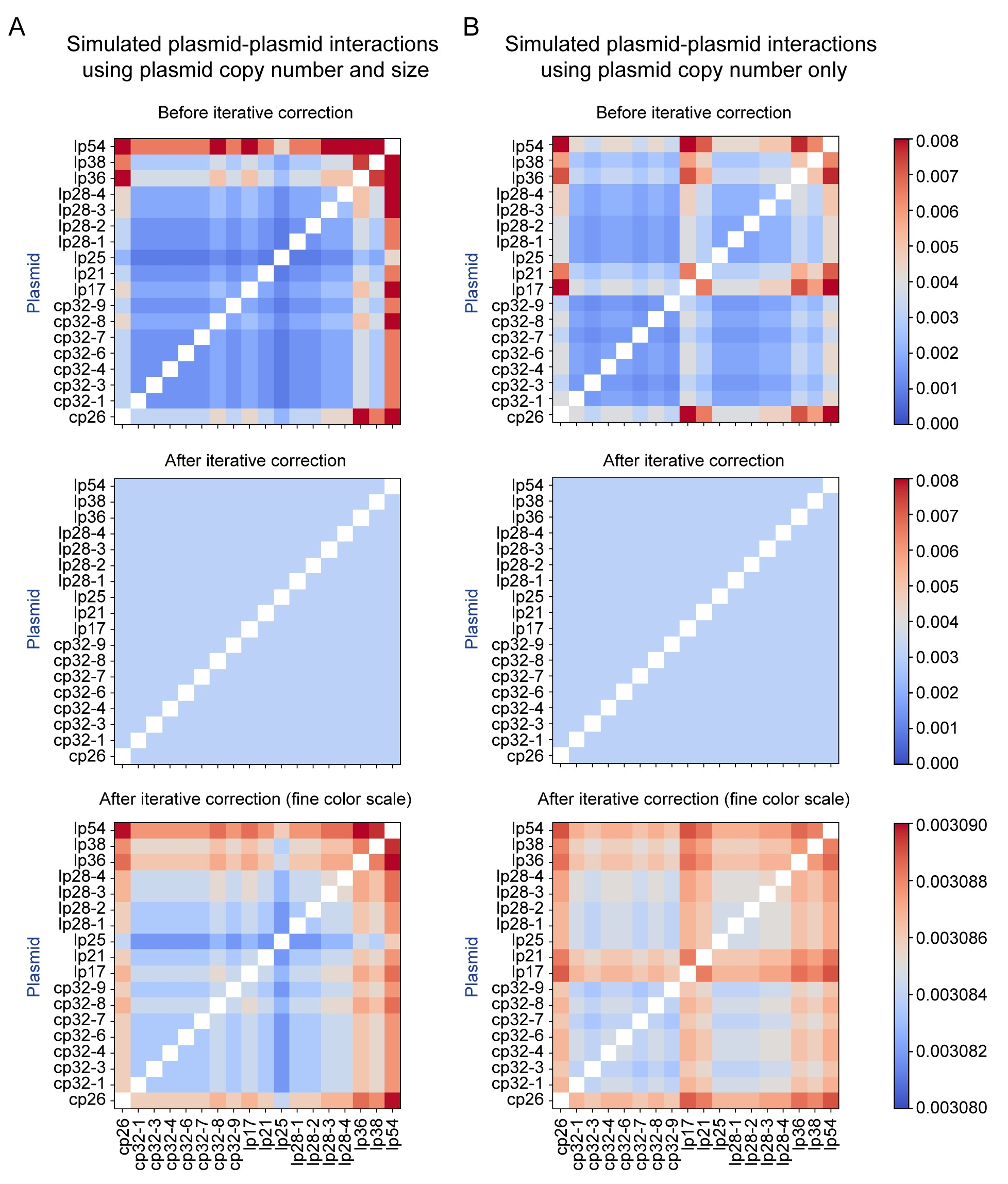

Supplement: S2 Fig — The contact probability between plasmids was simulated under the assumptions that plasmids are randomly interacting, independent of one another, and are “well mixed” within the cytoplasm (see Materials and methods). The calculation was performed accounting for plasmid copy numbers and plasmid lengths together (A) or only plasmid copy numbers (B). Top panels, the raw contact frequency expected between plasmids without normalization. Middle panels, the simulated contact frequency after normalization using iterative correction. Bottom panels, the same as middle panels, but shown with a much finer color scale. The color scales depicting contact frequencies in arbitrary units are shown at the right. We note that there is residual resemblance between bottom and top panels, and in the bottom panel, the row or column sums do not appear to be the same. This is because the iterative correction procedure stops when the row and column sums approach 1 within a pre-defined error tolerance (see Materials and methods), but not exactly at 1. (TIF) [file pgen.1010857.s002.tif]

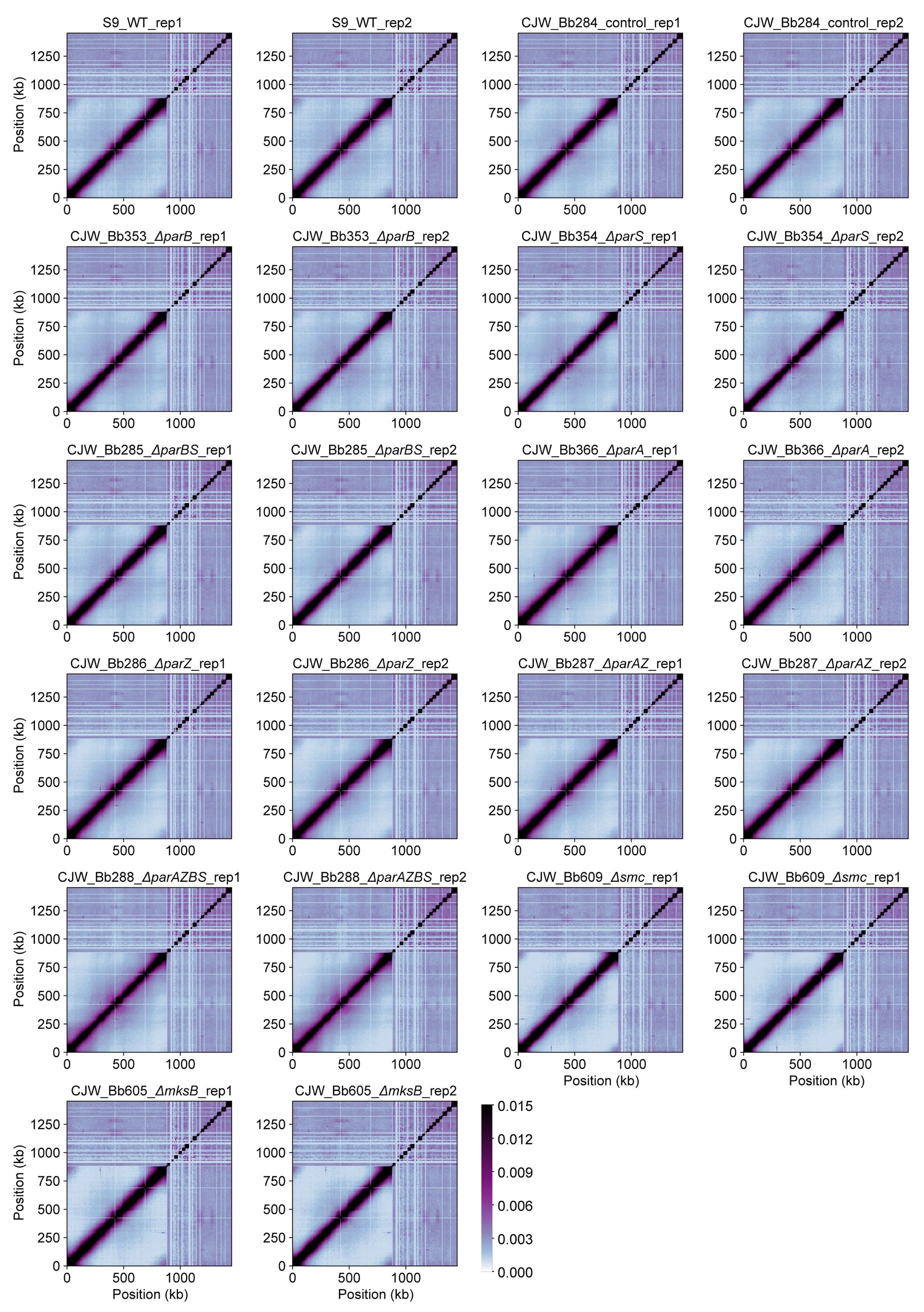

Supplement: S3 Fig — The normalized Hi-C interaction maps of all 22 experiments done for this study. The color scale depicting Hi-C interaction scores is shown at the bottom right. (TIF) [file pgen.1010857.s003.tif]

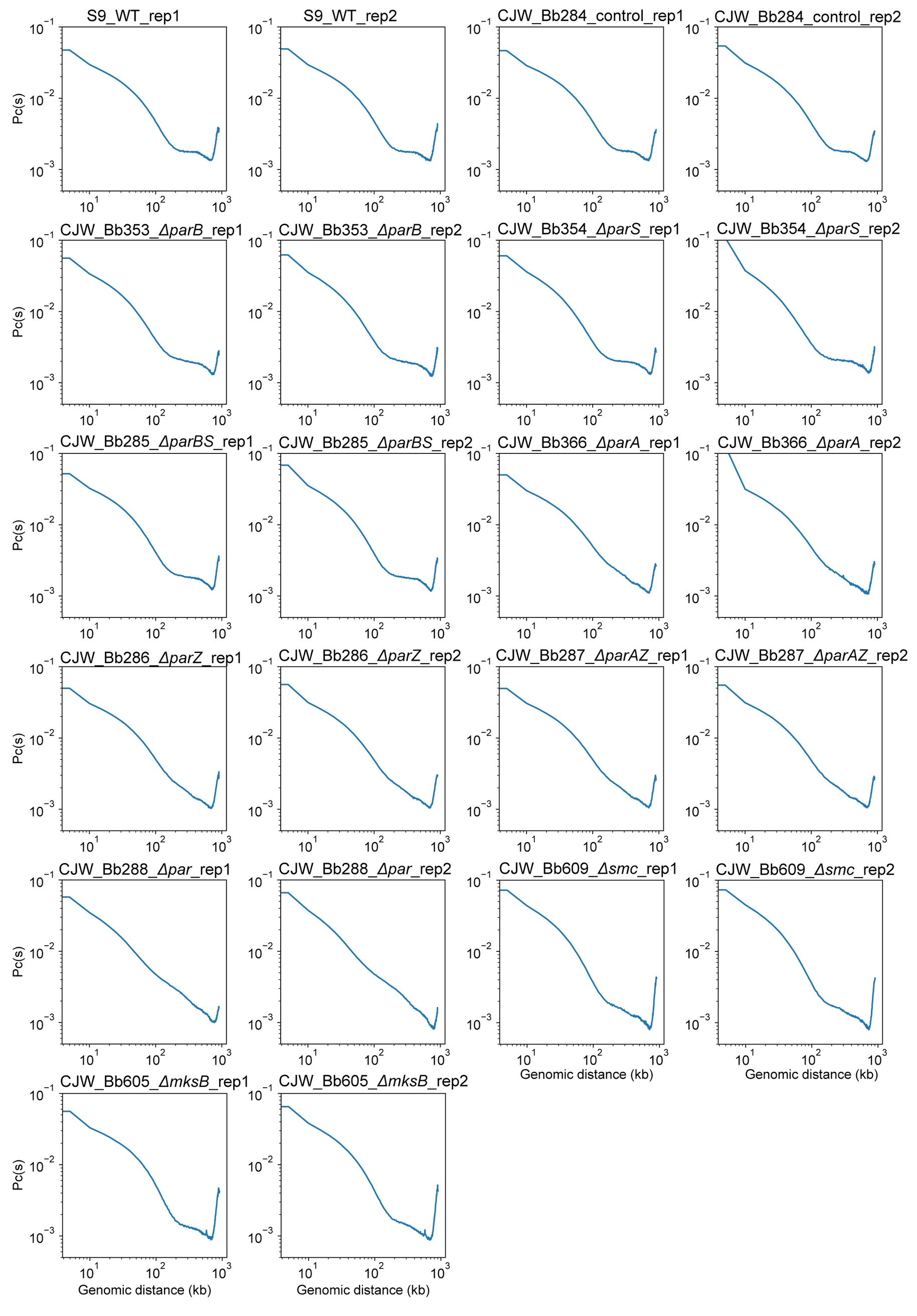

Supplement: S4 Fig — Pc(s) curves of all 22 Hi-C experiments done in this study. The x-axis indicates genomic distance while the y-axis shows averaged contact frequency. Only intra-chromosomal interactions were used to calculate the Pc(s) curves. (TIF) [file pgen.1010857.s004.tif]

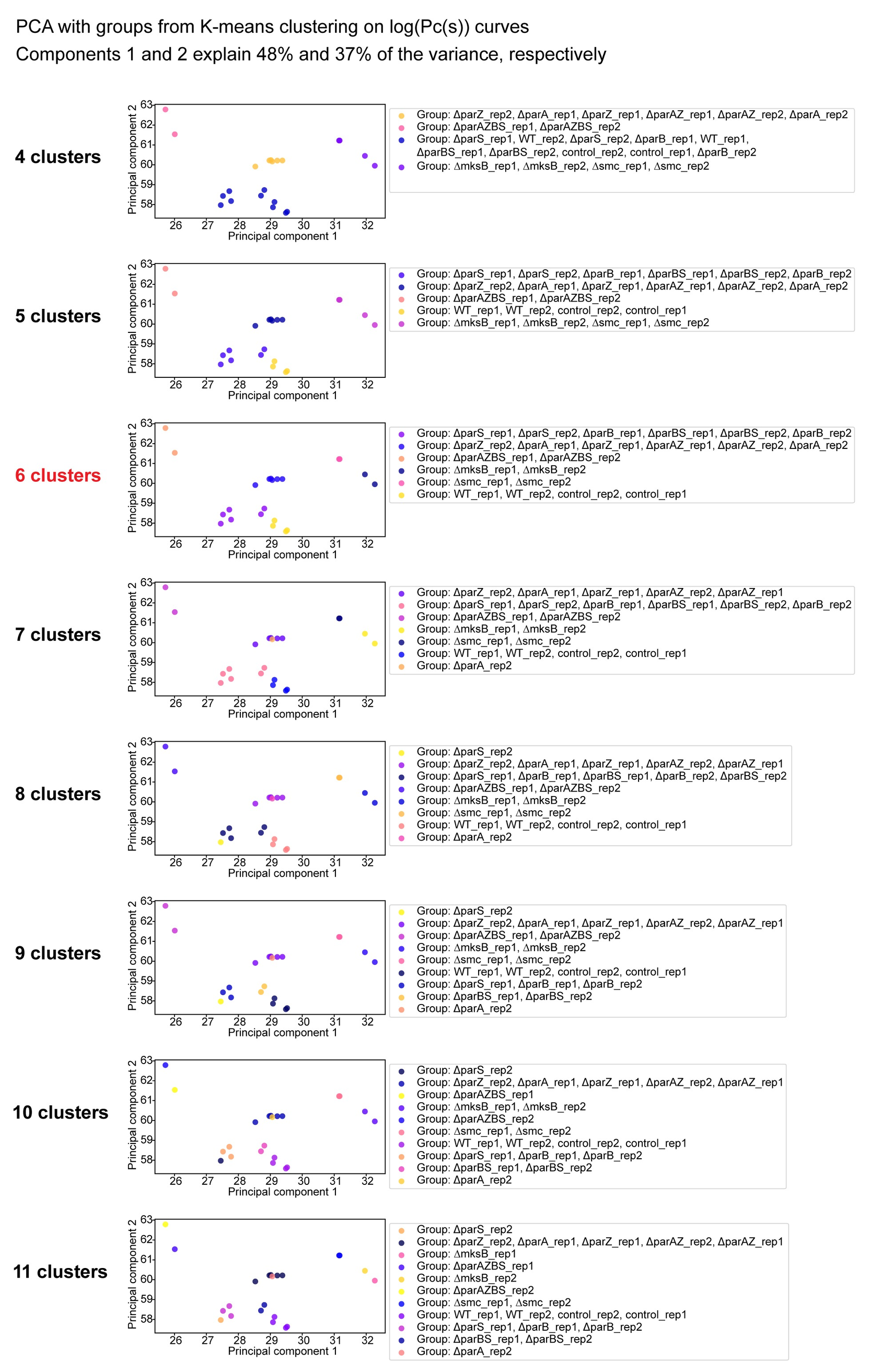

Supplement: S5 Fig — To better visualize the results of the k-means clustering generated by the Silhouette method, we performed Principal Component Analysis (PCA) and labeled the clustering results (see Materials and methods). The plots with up to six clusters gave nicely visually segregated groups. Beyond six, the two-dimensional projections from PCA showed poor segregation of the data points, and biological replicates were separated to different groups. (TIF) [file pgen.1010857.s005.tif]

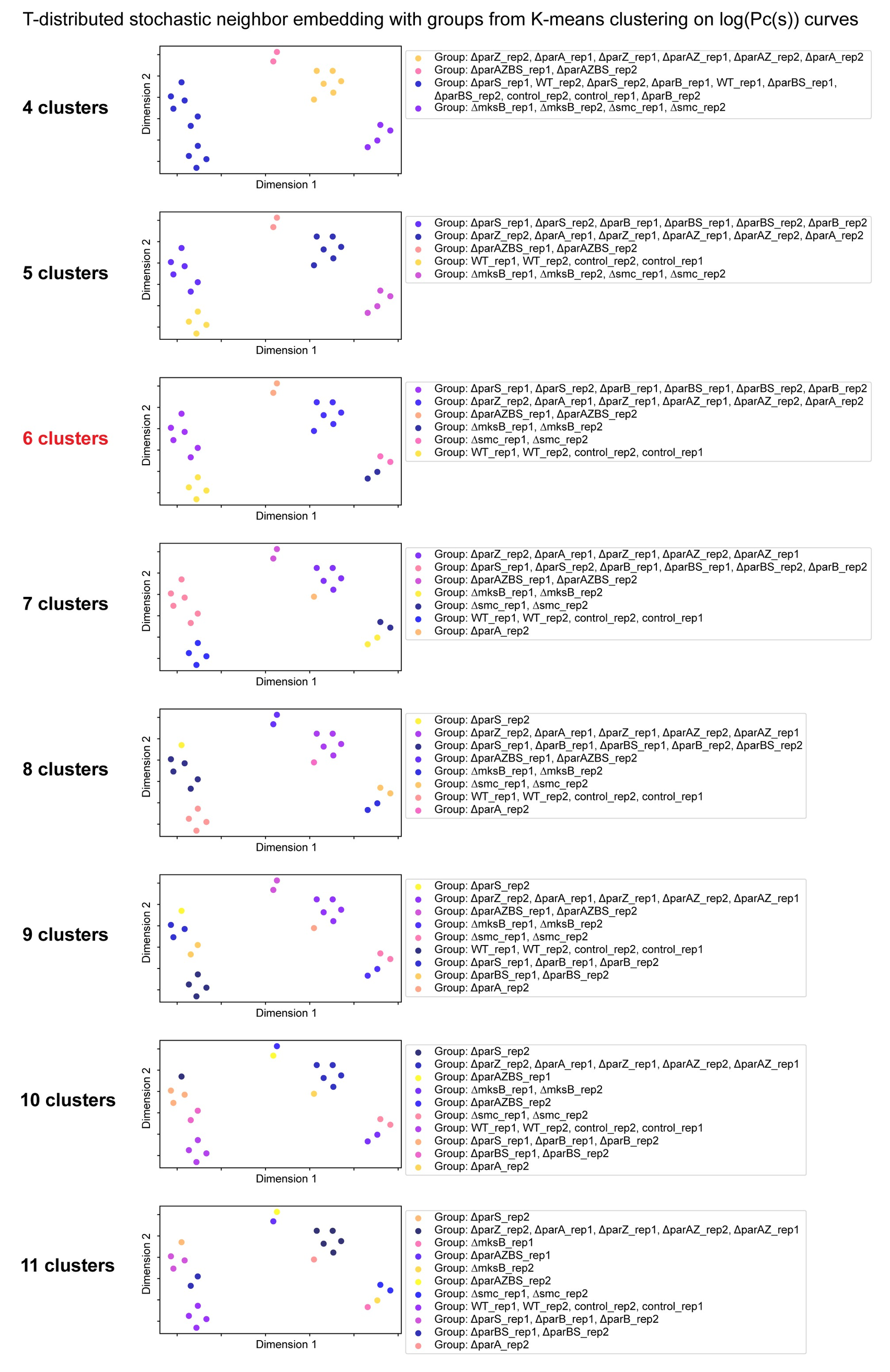

Supplement: S6 Fig — To better visualize the results of the k-means clustering generated by the Silhouette method, we performed t-distributed stochastic neighbor embedding (t-SNE) and labeled the clustering results (see Materials and methods). Similar to PCA, the plots with up to six clusters gave nicely visually segregated groups. Beyond six, the two-dimensional projections from t-SNE showed poor segregation of the data points, and biological replicates were separated to different groups. (TIF) [file pgen.1010857.s006.tif]

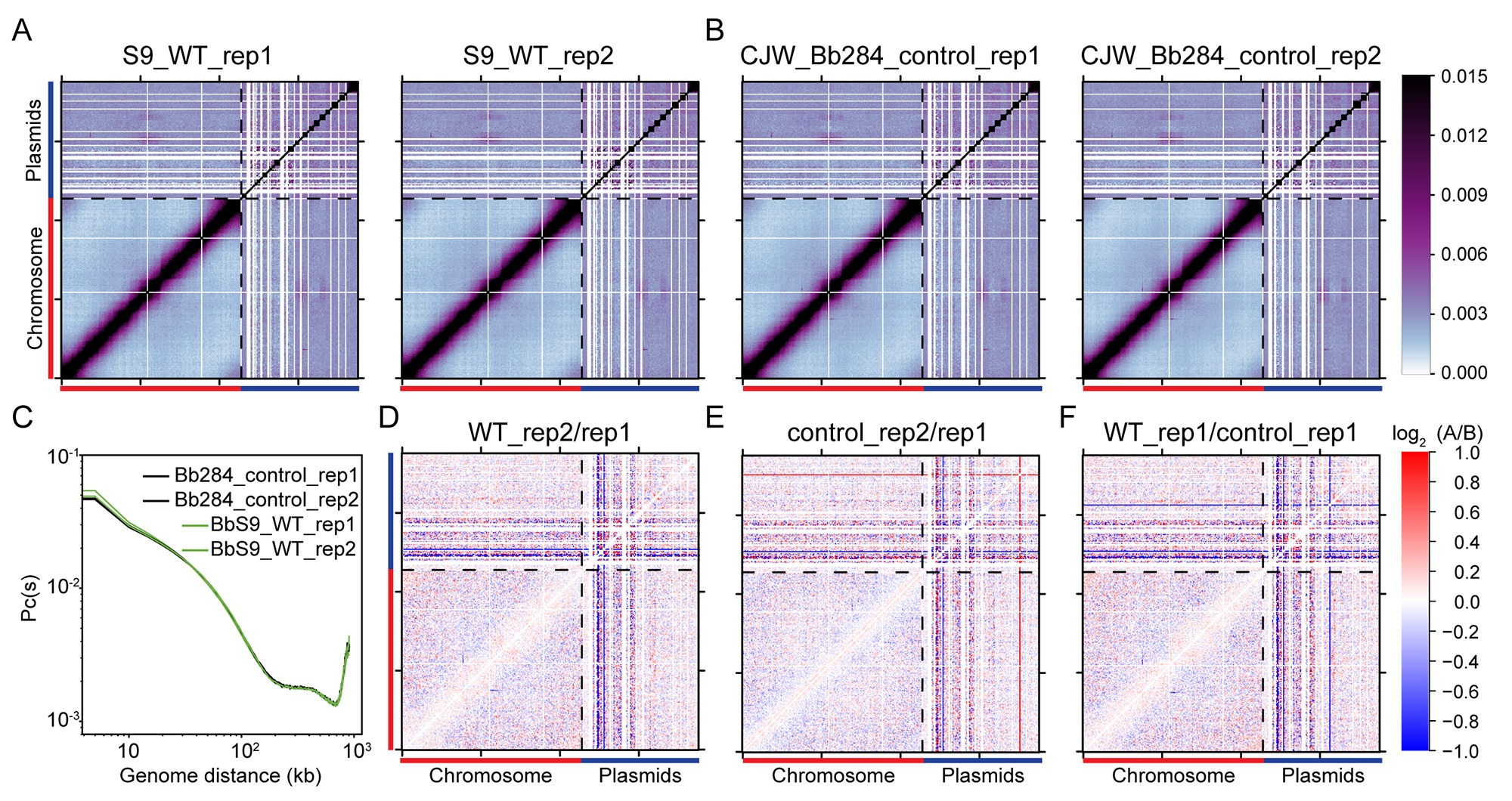

Supplement: S7 Fig — (A-B) Normalized Hi-C interaction maps of B. burgdorferi strains S9 (WT) and the control strain CJW_Bb284. Two biological replicates of each strain (rep1 and rep2) are shown. The color scale depicting Hi-C interaction scores in arbitrary units is shown at the right. (C) Pc(s) curves of the four samples. Pc(s) curves show the averaged contact frequency between all pairs of loci on the chromosome separated by set distance (s). The x-axis indicates the genomic distance of separation in kb. The y-axis represents the averaged contact frequency. The curves were computed for data binned at 5 kb. Only intra-chromosomal interactions were used to calculate the Pc(s) curves. (D-F) Log2 ratio plots comparing different Hi-C matrices. Log2(matrix 1/matrix 2) was calculated and plotted in the heatmaps. The identities of matrix 1/matrix 2 are shown at the top of each plot. The color scale is shown at the right of panel (F). (TIF) [file pgen.1010857.s007.tif]

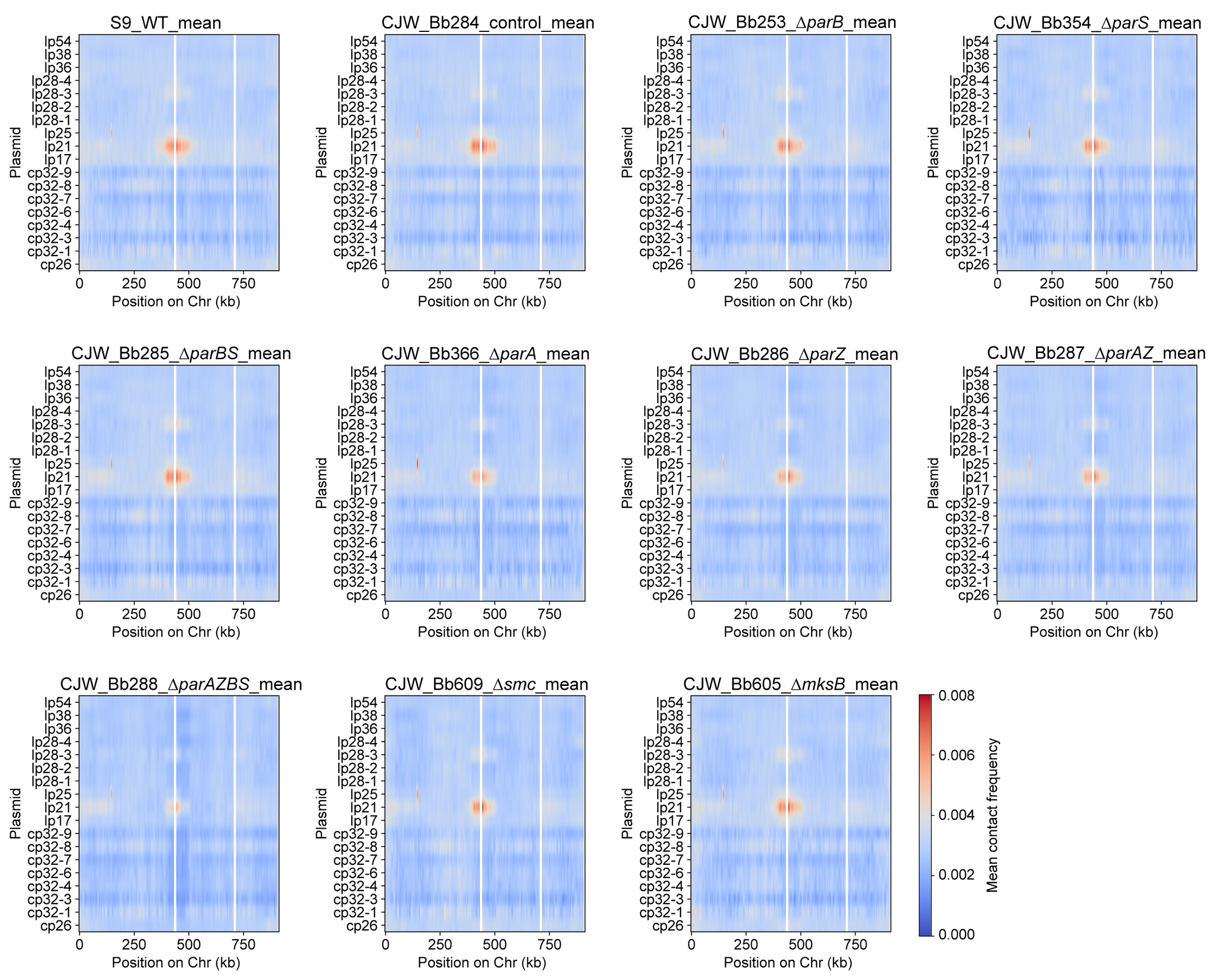

Supplement: S8 Fig — Calculated plasmid-chromosome interaction frequencies are shown. The x-axis shows chromosome location in kb. The y-axis specifies the different plasmids analyzed. The color indicates the contact frequency between each plasmid and chromosome locus. Each graph plots the mean value of the two biological replicates shown in S3 Fig. Data are binned at 5-kb resolution. The data were normalized including all the interactions in the genome (i.e. intra-chromosomal, plasmid-chromosome and plasmid-plasmid interactions). (TIF) [file pgen.1010857.s008.tif]

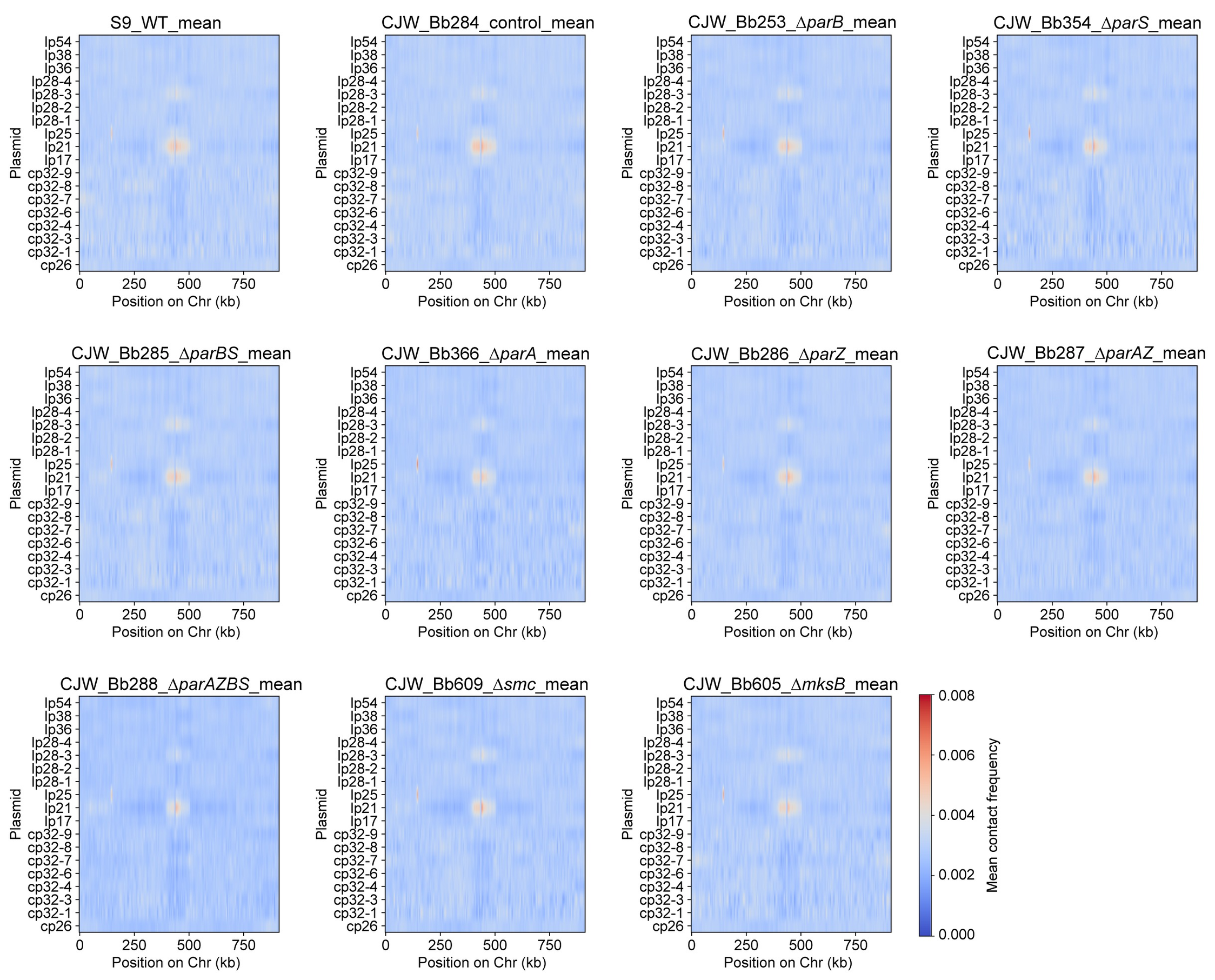

Supplement: S9 Fig — Plasmid-chromosome interactions from S8 Fig were renormalized using iterative correction to remove the influence of intra-chromosomal and plasmid-plasmid interactions (see Materials and methods). The data were normalized such that each row had the same total score, and each column had the same total score. (TIF) [file pgen.1010857.s009.tif]

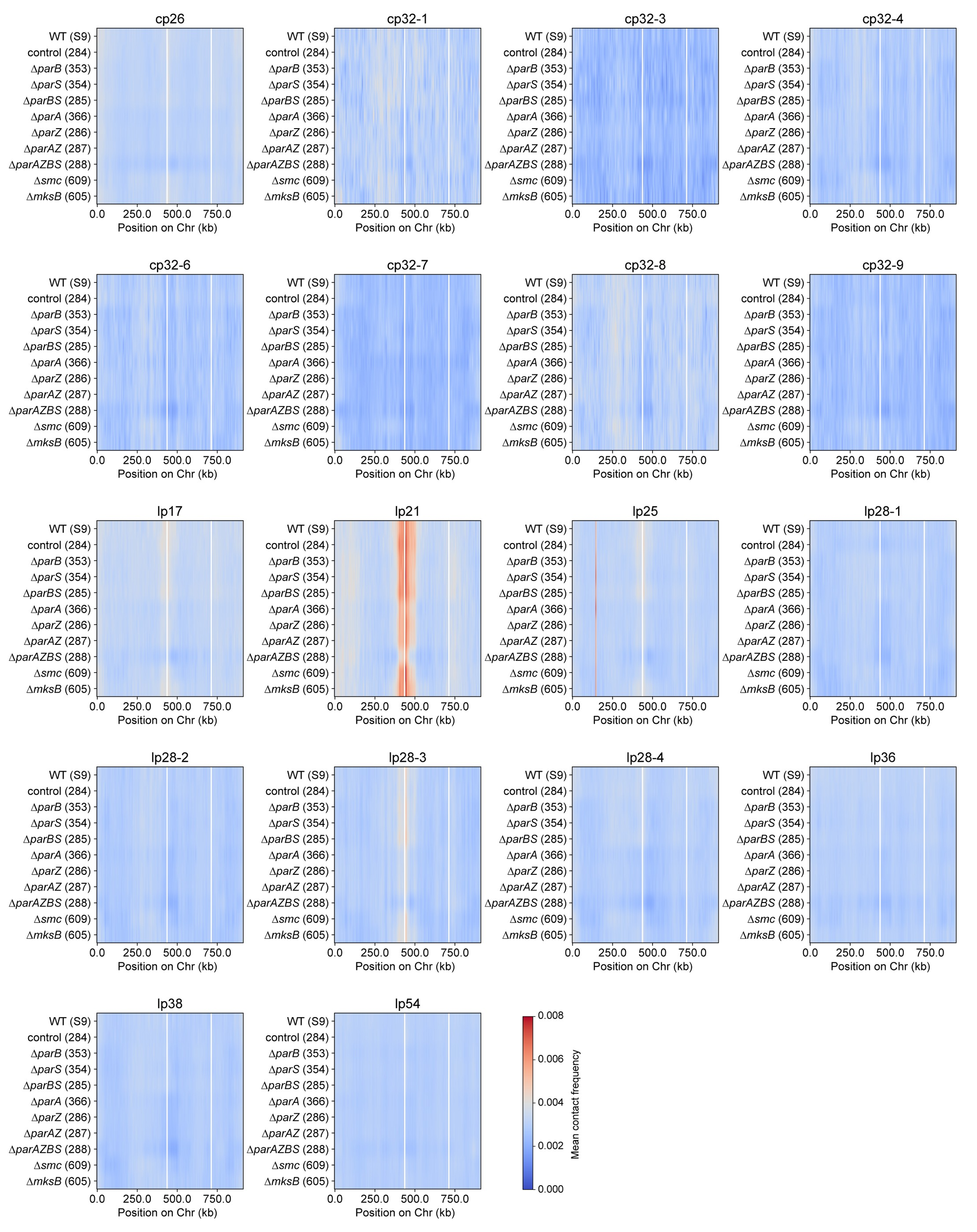

Supplement: S10 Fig — Calculated plasmid-chromosome interaction frequencies are shown. The x-axis shows the chromosome location in kb. The y-axis specifies the different mutants. The color indicates the contact frequency between each plasmid and chromosome locus. Each graph plots the mean value of the two biological replicates shown in S3 Fig. Data are binned at 5-kb resolution. (TIF) [file pgen.1010857.s010.tif]

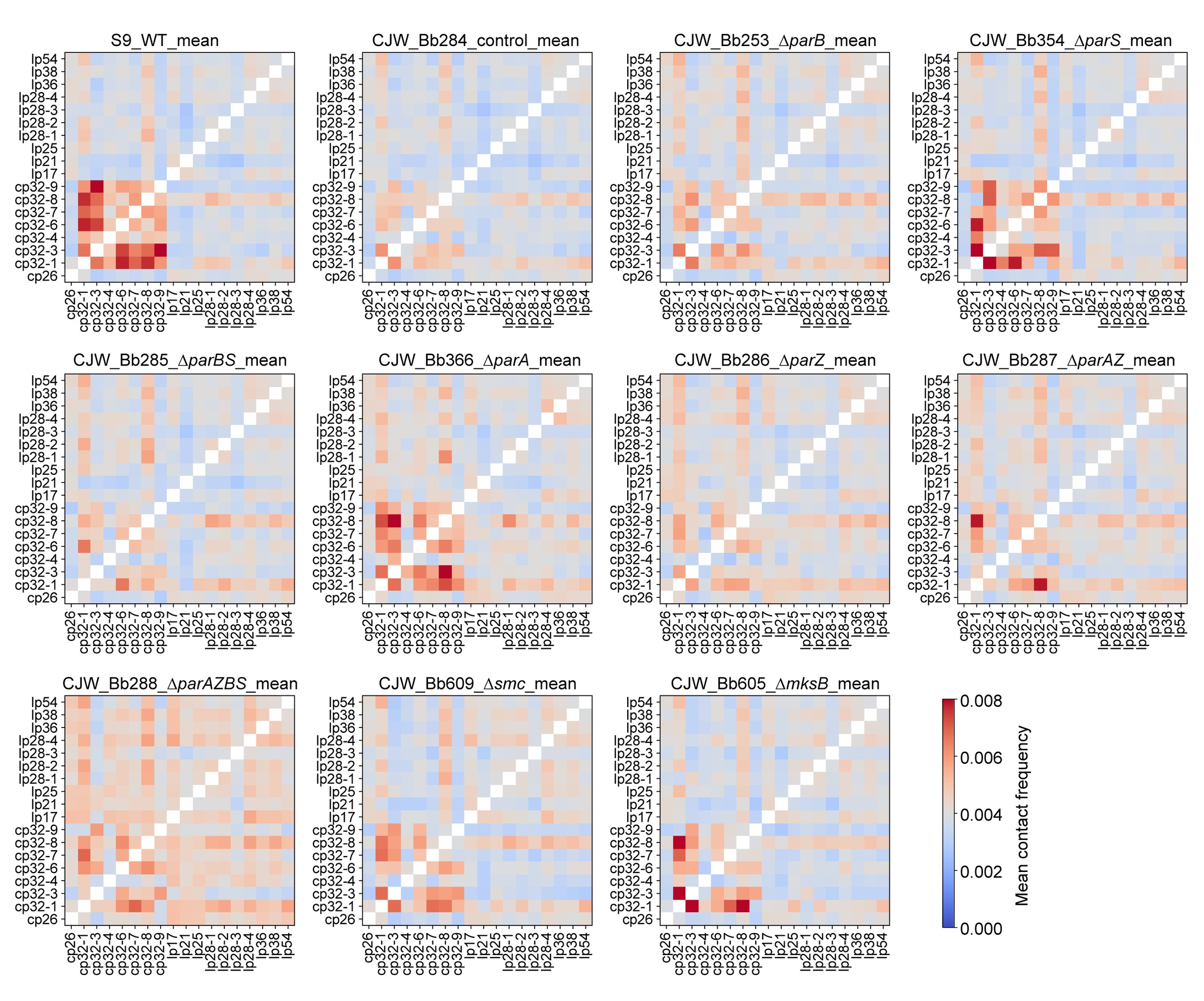

Supplement: S11 Fig — Calculated plasmid-plasmid contact frequencies in different strains. The x- and y-axes indicate the plasmids analyzed. The color shows the computed contact frequency. Each graph plots the mean of the two biological replicates shown in S3 Fig. The data were normalized including all the interactions in the genome (i.e. intra-chromosomal, plasmid-chromosome and plasmid-plasmid interactions). (TIF) [file pgen.1010857.s011.tif]

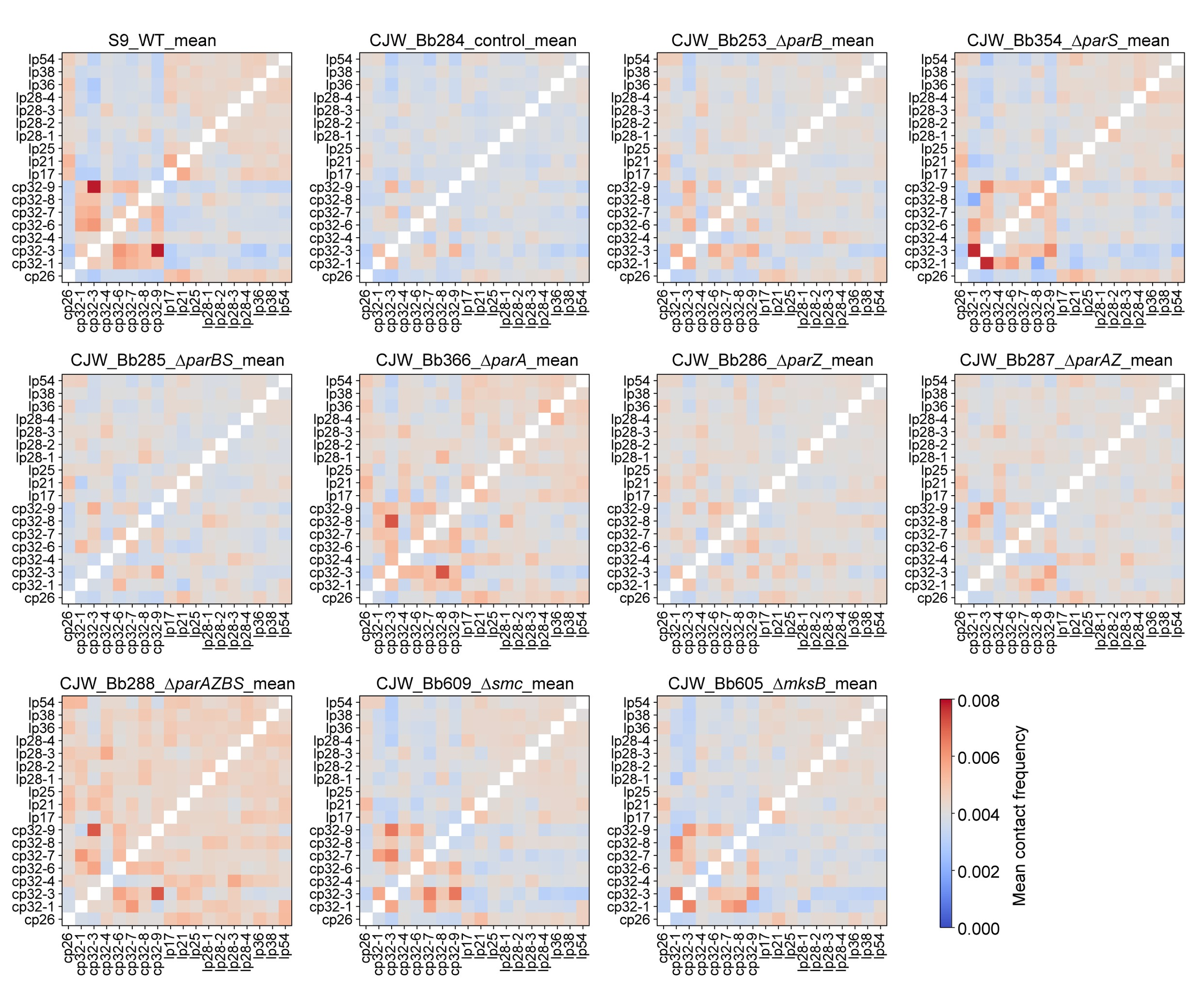

Supplement: S12 Fig — Plasmid-plasmid contact frequencies from S11 Fig were renormalized without plasmid-chromosome interactions. The data were normalized such that each row had the same total score, and each column had the same total score. (TIF) [file pgen.1010857.s012.tif]
