# Supplementary material for: Organization and replicon interactions within the highly segmented genome of Borrelia burgdorferi
Source: PLoS Genet. 2023 Jul 26;19(7):e1010857. doi: 10.1371/journal.pgen.1010857 (PMC10406323; doi:10.1371/journal.pgen.1010857)
Supplement: S1 Table — (DOCX) [file pgen.1010857.s013.docx]

**S1 Table. Bacterial strains used in this study.**

| **Strain** | **Genotype** | **Antibiotic resistance** | **Reference** | **Figure** |
| --- | --- | --- | --- | --- |
| S9 | Transformable derivative of the *B. burgdorferi* type strain B31; lacks endogenous plasmids cp9, lp5, and lp56; also known as B31-A3-68-*Δbbe02::PflaB-aadA* | Sr | [1] | 1-4, 5A-C, S1-S12 |
| CJW_Bb284 | S9-derived control strain; has gentamicin resistance cassette inserted between *parZ* and *parB* | Sr, Gm | [2] | 5A-C, 6A, 6D-I, S3-S12 |
| CJW_Bb285 | S9-derived *ΔparBS* strain | Sr, Gm | [2] | 5ABF, 7CF, S3-6, S8-12 |
| CJW_Bb286 | S9-derived *ΔparZ* strain | Sr, Gm | [2] | 5ABG, 7HL, S3-6, S8-12 |
| CJW_Bb287 | S9-derived *ΔparAZ* strain | Sr, Gm | [2] | 5ABG, 7IM, S3-6, S8-12 |
| CJW_Bb288 | S9-derived *ΔparAZBS* strain | Sr, Gm | [2] | 5ABH, 7JN, S3-6, S8-12 |
| CJW_Bb353 | S9-derived *ΔparB* strain | Sr, Gm | [2] | 5ABF, 7AD, S3-6, S8-12 |
| CJW_Bb354 | S9-derived *ΔparS* strain | Sr, Gm | [2] | 5ABF, 7BE, S3-6, S8-12 |
| CJW_Bb366 | S9-derived *ΔparA* strain | Sr, Km | [2] | 5ABG, 7GK, S3-6, S8-12 |
| CJW_Bb605 | S9-derived *ΔmksB* strain | Sr, Gm | This study | 5ABE, 6CFI, S3-6, S8-12 |
| CJW_Bb609 | S9-derived *Δsmc* strain | Sr, Gm | [2] | 5ABD, 6BEH, S3-6, S8-12 |

Sr, streptomycin resistance; Gm, gentamicin resistance; Km, kanamycin resistance.

**References**

1. Rego RO, Bestor A, Rosa PA. Defining the plasmid-borne restriction-modification systems of the Lyme disease spirochete Borrelia burgdorferi. J Bacteriol. 2011;193(5):1161-71. Epub 2011/01/05. doi: 10.1128/JB.01176-10. PubMed PMID: 21193609; PubMed Central PMCID: PMCPMC3067601.

2. Takacs CN, Wachter J, Xiang Y, Ren Z, Karaboja X, Scott M, et al. Polyploidy, regular patterning of genome copies, and unusual control of DNA partitioning in the Lyme disease spirochete. Nat Commun. 2022;13(1):7173. Epub 2022/12/01. doi: 10.1038/s41467-022-34876-4. PubMed PMID: 36450725; PubMed Central PMCID: PMCPMC9712426.
