# Supplementary material for: Organization and replicon interactions within the highly segmented genome of Borrelia burgdorferi
Source: PLoS Genet. 2023 Jul 26;19(7):e1010857. doi: 10.1371/journal.pgen.1010857 (PMC10406323; doi:10.1371/journal.pgen.1010857)
Supplement: S2 Table — (DOCX) [file pgen.1010857.s014.docx]

**S2 Table. Plasmids used in this study.**

| **Plasmid** | **Description** | **Reference** |
| --- | --- | --- |
| *pΔmksB(gent)* | Plasmid to make replace *∆mksB* with gentamycin resistance gene | This study |
| *pKIGent_parS^P1^_phoU* | Plasmid to insert *parS^P1^* near *phoU* | [1] |
| *pΔparA(kan)* | Plasmid to delete *parA* from *B. burgdorferi* chromosome | [1] |

**Reference**

1. Takacs CN, Wachter J, Xiang Y, Ren Z, Karaboja X, Scott M, et al. Polyploidy, regular patterning of genome copies, and unusual control of DNA partitioning in the Lyme disease spirochete. Nat Commun. 2022;13(1):7173. Epub 2022/12/01. doi: 10.1038/s41467-022-34876-4. PubMed PMID: 36450725; PubMed Central PMCID: PMCPMC9712426.
