# Supplementary material for: Organization and replicon interactions within the highly segmented genome of Borrelia burgdorferi
Source: PLoS Genet. 2023 Jul 26;19(7):e1010857. doi: 10.1371/journal.pgen.1010857 (PMC10406323; doi:10.1371/journal.pgen.1010857)
Supplement: S3 Table — (DOCX) [file pgen.1010857.s015.docx]

**S3 Table. Oligonucleotides used in this study.**

| **Oligo** | **Sequence** |
| --- | --- |
| NT968 | 5'-tggtaccgagctcggatccgggatttcttttgcgttgtttggtagatctactacatgtcc-3' |
| NT969 | 5'-ttttgtttttttacccgggcccgattgtcttaaaagaagtgtatcgaaattcaactcatg-3' |
| NT970 | 5'-cttcttttaagacaatcgggcccgggtaaaaaaacaaaagatcctttaaaggatcttttg-3' |
| NT971 | 5'-tatgccaatttgtcgcccgcggttcaaggaagatttcctattaaggttgaacttaagagc-3' |
| NT972 | 5'-aatcttccttgaaccgcgggcgacaaattggcataatttcccatgtttcttatttgaagg-3' |
| NT973 | 5'-ctctagatgcatgcattgcaataacccaaaaagatataaccgcaaaagacaataatatgc-3' |
| NT974 | 5'-tctttttgggttattgcaatgcatgcatctagagggcccaattcgccctatagtgagtcg-3' |
| NT975 | 5'-aaacaacgcaaaagaaatcccggatccgagctcggtaccaagcttgatgcatagcttgag-3' |
