# Supplementary material for: Organization and replicon interactions within the highly segmented genome of Borrelia burgdorferi
Source: PLoS Genet. 2023 Jul 26;19(7):e1010857. doi: 10.1371/journal.pgen.1010857 (PMC10406323; doi:10.1371/journal.pgen.1010857)
Supplement: S4 Table — (DOCX) [file pgen.1010857.s016.docx]

**S4 Table. Next generation sequencing samples used in this study**.

| **Sample name** | **Figure** | **Reference** | **Identifier** | **Numbers of nonduplicated double-side unique mapped reads** |
| --- | --- | --- | --- | --- |
| HiC_CJW_Bb284_rep1 | 5A-C, 6A, 6D-I, S3-S12 | This study | [GSM7056005](https://www.ncbi.nlm.nih.gov/geo/query/acc.cgi?acc=GSM7056005) | 12,949,960 |
| HiC_CJW_Bb284_rep2 | 5A-C, 6D-I, S3-S12 | This study | [GSM7056006](https://www.ncbi.nlm.nih.gov/geo/query/acc.cgi?acc=GSM7056006) | 18,388,642 |
| HiC_CJW_Bb285_rep1 | 5ABF, 7CF, S3-6, S8-12 | This study | [GSM7056007](https://www.ncbi.nlm.nih.gov/geo/query/acc.cgi?acc=GSM7056007) | 15,384,363 |
| HiC_CJW_Bb285_rep2 | 5ABF, S3-6, S8-12 | This study | [GSM7056008](https://www.ncbi.nlm.nih.gov/geo/query/acc.cgi?acc=GSM7056008) | 17,196,945 |
| HiC_CJW_Bb286_rep1 | 5ABG, 7HL, S3-6, S8-12 | This study | [GSM7056009](https://www.ncbi.nlm.nih.gov/geo/query/acc.cgi?acc=GSM7056009) | 16,232,326 |
| HiC_CJW_Bb286_rep2 | 5ABG, S3-6, S8-12 | This study | [GSM7056010](https://www.ncbi.nlm.nih.gov/geo/query/acc.cgi?acc=GSM7056010) | 18,775,364 |
| HiC_CJW_Bb287_rep1 | 5ABG, 7IM, S3-6, S8-12 | This study | [GSM7056011](https://www.ncbi.nlm.nih.gov/geo/query/acc.cgi?acc=GSM7056011) | 14,421,014 |
| HiC_CJW_Bb287_rep2 | 5ABG, S3-6, S8-12 | This study | [GSM7056012](https://www.ncbi.nlm.nih.gov/geo/query/acc.cgi?acc=GSM7056012) | 16,560,484 |
| HiC_CJW_Bb288_rep1 | 5ABH, 7JN, S3-6, S8-12 | This study | [GSM7056013](https://www.ncbi.nlm.nih.gov/geo/query/acc.cgi?acc=GSM7056013) | 15,605,994 |
| HiC_CJW_Bb288_rep2 | 5ABH, S3-6, S8-12 | This study | [GSM7056014](https://www.ncbi.nlm.nih.gov/geo/query/acc.cgi?acc=GSM7056014) | 17,741,544 |
| HiC_CJW_Bb353_rep1 | 5ABF, 7AD, S3-6, S8-12 | This study | [GSM7056015](https://www.ncbi.nlm.nih.gov/geo/query/acc.cgi?acc=GSM7056015) | 15,306,050 |
| HiC_CJW_Bb353_rep2 | 5ABF, S3-6, S8-12 | This study | [GSM7056016](https://www.ncbi.nlm.nih.gov/geo/query/acc.cgi?acc=GSM7056016) | 13,407,970 |
| HiC_CJW_Bb354_rep1 | 5ABF, 7BE, S3-6, S8-12 | This study | [GSM7056017](https://www.ncbi.nlm.nih.gov/geo/query/acc.cgi?acc=GSM7056017) | 13,917,027 |
| HiC_CJW_Bb354_rep2 | 5ABF, S3-6, S8-12 | This study | [GSM7056018](https://www.ncbi.nlm.nih.gov/geo/query/acc.cgi?acc=GSM7056018) | 11,211,083 |
| HiC_CJW_Bb366_rep1 | 5ABG, 7GK, S3-6, S8-12 | This study | [GSM7056019](https://www.ncbi.nlm.nih.gov/geo/query/acc.cgi?acc=GSM7056019) | 15,375,430 |
| HiC_CJW_Bb366_rep2 | 5ABG, S3-6, S8-12 | This study | [GSM7056020](https://www.ncbi.nlm.nih.gov/geo/query/acc.cgi?acc=GSM7056020) | 10,765,224 |
| HiC_CJW_Bb605_rep1 | 5ABE, 6I, S3-6, S8-12 | This study | [GSM7056021](https://www.ncbi.nlm.nih.gov/geo/query/acc.cgi?acc=GSM7056021) | 8,440,508 |
| HiC_CJW_Bb605_rep2 | 5ABE, 6CFI, S3-6, S8-12 | This study | [GSM7056022](https://www.ncbi.nlm.nih.gov/geo/query/acc.cgi?acc=GSM7056022) | 9,953,656 |
| HiC_CJW_Bb609_rep1 | 5ABD, 6BEH, S3-6, S8-12 | This study | [GSM7056023](https://www.ncbi.nlm.nih.gov/geo/query/acc.cgi?acc=GSM7056023) | 9,798,914 |
| HiC_CJW_Bb609_rep2 | 5ABD, 6H, S3-6, S8-12 | This study | [GSM7056024](https://www.ncbi.nlm.nih.gov/geo/query/acc.cgi?acc=GSM7056024) | 11,279,804 |
| HiC_CJW_S9WT_rep1 | 1-4, 5A-C, S1-S12 | This study | [GSM7056025](https://www.ncbi.nlm.nih.gov/geo/query/acc.cgi?acc=GSM7056025) | 10,377,706 |
| HiC_CJW_S9WT_rep2 | 5A-C, S1-S12 | This study | [GSM7056026](https://www.ncbi.nlm.nih.gov/geo/query/acc.cgi?acc=GSM7056026) | 11,847,047 |
